# Hippocampals neurogenesis is impaired in mice with a deletion in the coiled coil domain of Talpid3—implications for Joubert syndrome

**DOI:** 10.1093/hmg/ddac095

**Published:** 2022-04-26

**Authors:** Andrew L Bashford, Vasanta Subramanian

**Affiliations:** Department of Biology and Biochemistry, University of Bath, Bath BA2 7AY, UK; Department of Biology and Biochemistry, University of Bath, Bath BA2 7AY, UK

## Abstract

Mutations in Talpid3, a basal body protein essential for the assembly of primary cilia, have been reported to be causative for Joubert Syndrome (JS). Herein, we report prominent developmental defects in the hippocampus of a conditional knockout mouse lacking the conserved exons 11 and 12 of *Talpid3*. At early postnatal stages, the *Talpid3* mutants exhibit a reduction in proliferation in the dentate gyrus and a disrupted glial scaffold. The occurrence of mis-localized progenitors in the granule cell layer suggests a role for the disrupted glial scaffold in cell migration resulting in defective subpial neurogenic zone-to-hilar transition. Neurospheres derived from the hippocampus of *Talpid3^fl/fl^UbcCre* mouse, in which *Talpid3* was conditionally deleted, lacked primary cilia and were smaller in size. In addition, neurosphere cells showed a disrupted actin cytoskeleton and defective migration. Our findings suggest a link between the hippocampal defects and the learning/memory deficits seen in JS patients.

## Introduction

Primary cilia are highly conserved sensory organelles, which protrude from the cell surface like an antenna. They are non-motile and have an axoneme made of microtubules (9 + 0) and a basal body that acts as a microtubule organizing centre (1). Motor proteins facilitate the movements of numerous proteins to and from the primary cilium. In addition, the primary cilium acts as a specialized compartment with a unique environment and the presence of transmembrane receptors allows it to transduce extracellular signals (2). Primary cilia are essential for transduction of signals of the sonic hedgehog (Shh) pathway in vertebrates as lack of cilia leads to abrogation of Shh signalling (3).

There are a number of rare human conditions, in which cilia are disrupted collectively termed ‘ciliopathies’. These syndromes often involve a number of organs such as the brain, limbs, kidneys and eyes (4,5). One such ciliopathy is Joubert syndrome (JS) (6,7). Several causative genes have been identified for JS and currently numbering at 34 (8). One recently identified gene with mutations in JS patients is *KIAA0586* or *TALPID33* (9–12).

*Talpid3* was first identified in the *talpid* chick mutant (13,14). Subsequently, it was found that the *talpid* chick embryos with a mutation in *Talpid3* lack primary cilia leading to aberrant Shh signalling (15). Talpid3 is a centrosomal protein essential for the docking of the basal body to the cell membrane (16). The Talpid3 protein has a conserved coiled coil domain and rescue assays with expression vectors encoding this domain alone showed that it is essential but not sufficient to rescue the formation of cilia and Shh signalling in the neural tube of the *Talpid3* chick (16). *Talpid3* is conserved in vertebrates and is essential for cilia formation not only in the chick (16) but also in the mice (17) and zebrafish (18). Mice with a constitutive deletion of the conserved exons 11 and 12 of *Talpid3* (*2700049A03Rik*) are embryonic lethal. Conditional deletion of these exons in the limb leads to polydactyly, which was attributed to loss of primary cilia and aberrant Shh signalling (17).

Neural defects seen in JS often include hydrocephaly and hypoplasia or aplasia of the cerebellar vermis and the classic molar tooth sign. Another phenotype commonly described in JS is intellectual disability (19). Hippocampal abnormalities such as malrotation have also been reported in a subset of JS subjects suggesting a potential link with the learning and memory deficits seen in these patients (20,21). This aspect of JS is not well studied mainly due to the paucity of viable mouse models that fully recapitulate the JS brain phenotype.

The mouse hippocampus consists of two distinct structures—the cornu ammonius (CA1, Ca2 and CA3) and the dentate gyrus (DG. The dentate gyrus of the hippocampus is one of the few regions of the CNS in which neurogenesis continues into adulthood (22–24). A number of signalling pathways regulate the development of the dentate gyrus. One well studied pathway is the Shh pathway, which has been shown to be required for the proliferation of granule progenitors (25,26) and transduction of Shh signalling through the primary cilium has been shown to be important for hippocampal development. Conditional deletion of genes essential for Shh signalling, such as *Smo*, leads to abnormal development of neuronal precursors and causes hypotrophy of the dentate gyrus in mice (26). Mice lacking cilia components, such as *Kif3a* (a member of the kinesin family), *Ift88* (intraflagellar transport component) and *B9d2* (basal body component) all show defects in adult hippocampal neural precursors (26,27).

We have generated mice with a conditional deletion of *Talpid3* in the central nervous system by crossing the *Talpid3^fl/fl^* mouse with a *NestinCre* deleter mouse strain (28). The *Talpid3^fl/fl^ NesCre* mice exhibited ataxia and examination of the brain showed a strikingly hypoplastic cerebellum with hall mark features of JS (29). Since primary cilia and Shh signalling play an important role in stem cell proliferation in the dentate gyrus and *Talpid3* is essential for the formation of primary cilia, we examined the hippocampus of *Talpid3* JS mice for defects that may have bearing on the hippocampal defects seen in JS patients.

## Results

We undertook a detailed analysis of the hippocampus of the *Talpid3^fl/fl^ NesCre* (*Talpid3* mutant) and the *Talpid3^fl/fl^* (control) siblings between e18.5 and P15. The earliest stage at which the different regions of the mouse hippocampus can be clearly identified is e18.5. The pyramidal neurons of Ammon’s horn complete their migration by P5 and the subpial neurogenic zone (SPZ) to hilar transition can be seen at this stage. These structures mature between P10 and P15 accompanied by growth and proliferation of the subgranular zone (SGZ) of the dentate gyrus. This is followed by a decrease in the rate of proliferation after P15. The oldest time point at which we investigated the hippocampus of the *Talpid3* mutant mice was P15 due to the development of extreme ataxia at which point the mice have to be culled.

### The *Talpid3 mutant* hippocampus lacks primary cilia

**Figure 1:**
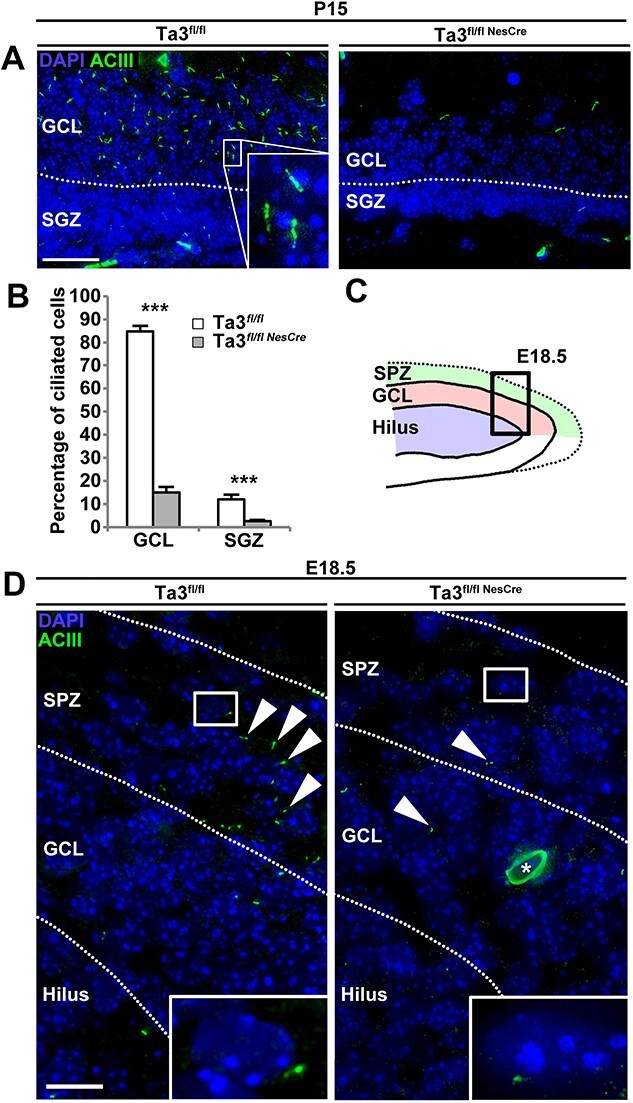
The *Talpid3* mutant hippocampus lacks primary cilia. (**A**) P15 dentate gyrus immuno-stained with anti-ACIII antibody. Primary cilia are seen primarily in the GCL of the DG in the wildtype (Ta3^fl/fl^). Mutant (Ta3^fl/flNesCre^) DG exhibits a striking loss of primary cilia. (**B**) Quantification of ciliated cells in the SGZ and GCL. Mutant DG (Ta3^fl/flNesCre^) shows a significant reduction in both regions. (**C**) Illustration of 18.5 dentate gyrus with box indicating field of view in (**D**). (D) E18.5 dentate gyrus immuno-stained with anti-ACIII antibody. In the control (Ta3^fl/fl^), primary cilia are found primarily in the SPZ. Mutants show a striking loss of primary cilia in all regions. Scale 25 μm (A, D). Error bars: (B) SEM (*n* = 3), ^***^P ≤ 0.001 Student’s *t*-test. Single asterisk in (D) indicates autofluorescence.

We immunostained sections of the brain from the *Talpid3* mutant and control mice with antibody to Adenyl cyclase 3 (ASCIII) (30) to detect primary cilia (Fig. 1). At P15, primary cilia were present in most cells in the predominantly quiescent granule cell layer (GCL) of the control dentate gyrus but were not detectable in the GCL of *Talpid3* mutant mice (Fig. 1A and B). Compared to control siblings, the SGZ of the *Talpid3* mutant mice also showed a significant reduction (80%) in the number of cells with primary cilia. We also immuno-stained for primary cilia at e18.5, when the hilus, GCL and SPZ are identifiable in the dentate gyrus (Fig. 1C). At e18.5, primary cilia were clearly seen in the SPZ (Fig. 1D) of the sibling control mice but were absent in the *Talpid3* (Fig. 1D).

### The *Talpid3* mutant hippocampus is misshapen with defects in the dentate gyrus

**Figure 2:**
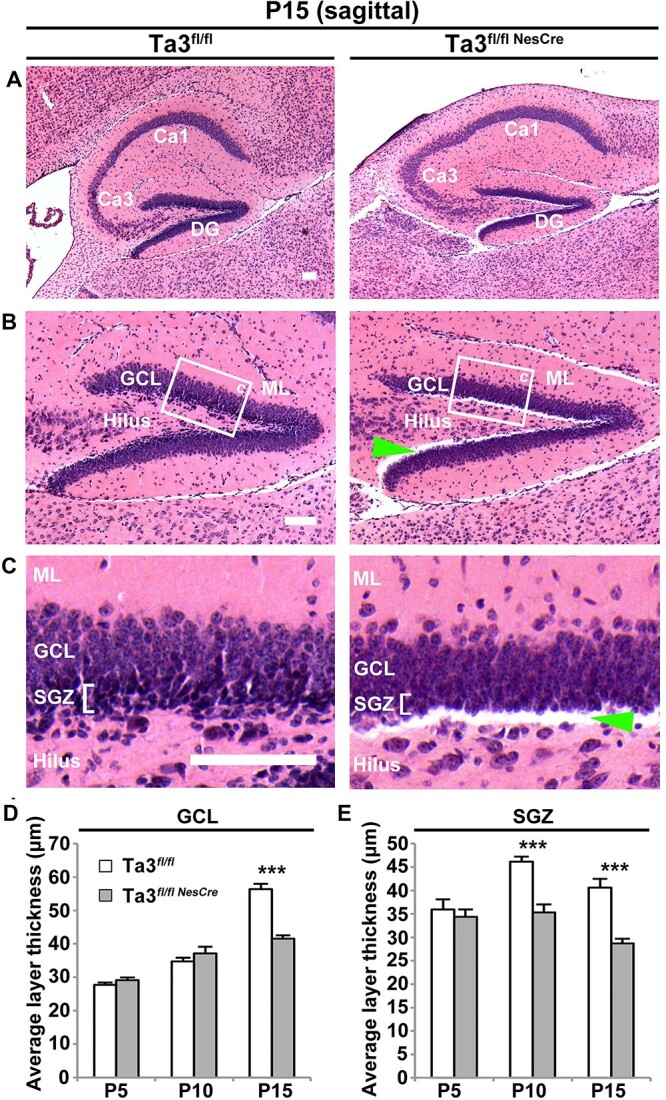
*Talpid3* mutant hippocampus has a thinner SGZ and GCL. (**A**) Sagittal sections stained with haematoxylin and eosin showing the dorsal hippocampus. Mutant hippocampus (Ta3^fl/flNesCre^) has a more elliptical shape but the gross organization of Ammon’s horn and the DG is similar to control (Ta3^fl/fl^). (**B**) Higher magnification of DG. Note the thinner GCL and a loss of tissue integrity at the SGZ/hilus boundary in the mutant (Ta3^fl/flNesCre^). (**C**) Higher magnification of GCL. Mutants (Ta3^fl/flNesCre^) show a loss of darkly pigmented cells in the SGZ. (**D**) Quantification of GCL thickness for P5, P10, P15 mice. P15 mutants (Ta3^fl/flNesCre^) show a reduction in GCL thickness. (**E**) Quantification of SGZ thickness of the hippocampus in P5, P10, P15 mice. P10 and P15 mutants (Ta3^fl/flNesCre^) show a reduction in GCL thickness. Arrow indicates loss of tissue integrity. Scale bars: 100 μm (A–D). Error bars: (D, E) SEM (*n* = 3), ^***^P ≤ 0.001, Student’s *t*-test.

The morphology of the hippocampus at e18.5 was comparable between control and *Talpid3* mutant mice but by early postnatal stages, defects in the hippocampus were noticeable. On first inspection, the P15 *Talpid3* mutant hippocampus in H&E stained sections appeared more elliptical and compressed as compared to that in the control sibling mice, presumably a consequence of the hydrocephaly (Fig. 2A). The gross morphology of the dentate gyrus and Ammon’s horn appeared similar to control sibling mice. However, upon closer examination, the dentate gyrus appeared thinner with a lower cell density (Fig. 2C). At P15, both the GCL and the SGZ of the dentate gyrus were thinner in the *Talpid3* mutant (Fig. 2D and E). The SGZ of the *Talpid3* mutant mice also had a reduced number of darkly pigmented cells (Fig. 2B and C), which have previously been identified as the progenitor population (26,31). The region below the SGZ also consistently exhibited a loss of tissue integrity which often resulted in tearing of the section (Fig. 2B and C).

### Organization of the Hippocampus

**Figure 3:**
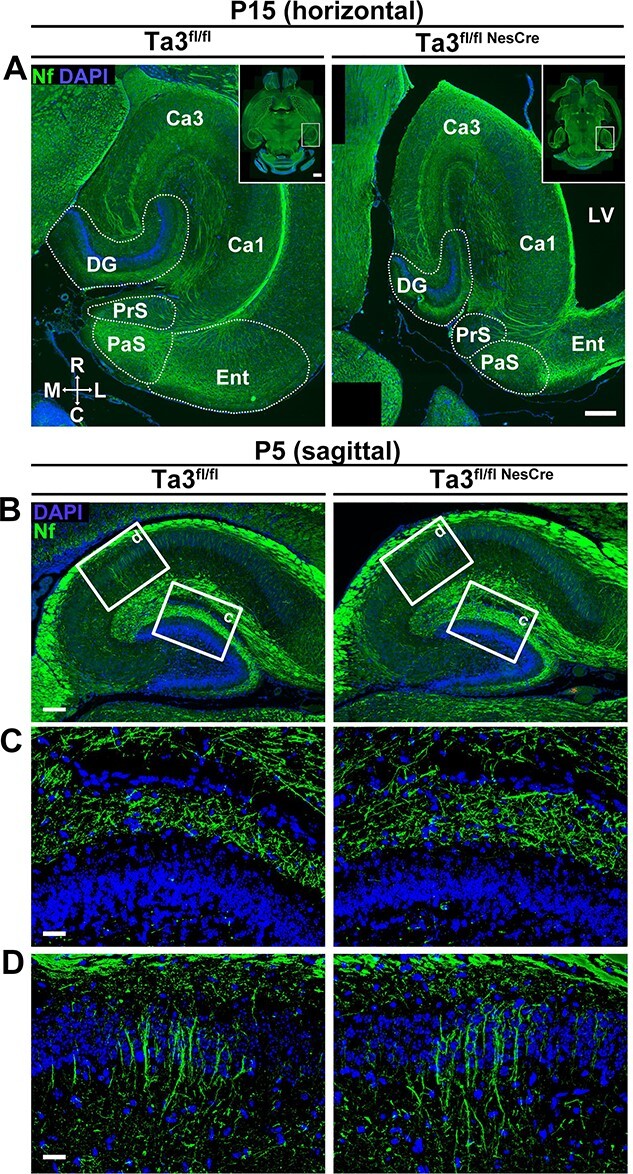
Mutant (Ta3^fl/flNesCre^) hippocampus is misshapen but with similar axonal organization. (**A**) Horizontal P15 sections of the hippocampus immuno-stained for neurofilament (165 kDa). The mutant (Ta3^fl/flNesCre^) hippocampus is elliptical with a smaller dentate gyrus. The presubiculum (PrS) and parasubiculum (PaS) are reduced in size. Compass indicates orientation; C, caudal, L, lateral, M, medial, R, Rostral. (**B**) Sagittal P5 sections stained for neurofilament. Boxed areas indicate regions of higher magnification. (**C**) Axons of the performant pathway terminating in the ML and (**D**) radial axons of the CA1 field are similar between control and mutant. Scale bar: 250 μm (A), 100 μm (B), 25 μm.

Mature axonal tracts were identified by immuno-labelling for neurofilament (165 kDa). In horizontal sections of the P15 *Talpid3* mutant brain, the hippocampus was seen to be more elliptical as compared to the control (Fig. 3A) and the dentate gyrus was smaller. Although the GCL displayed the characteristic ‘U’ shape, its thickness was reduced compared to the control GCL. Despite the reduced thickness of the dentate gyrus, both the CA1 and CA3 fields in the *Talpid3* mutant were comparable in size to the control (Fig. 3A). However, structures caudal to the hippocampus such as the presubiculum (PrS) and parasubiculum (PaS) were smaller in the *Talpid3* mutant (Fig. 3A).

Many of the key axonal connections in the hippocampus are established by P5. To assess the effect of the loss of *Talpid3* on this, we analyzed sagittal sections of the P5 brain immuno-stained for neurofilament (165 kDa) (Fig. 3B). Axons from the perforant path terminate in the molecular layer (ML) above the GCL. Both control and *Talpid3* mutant showed a similar density and branching of axonal tracts (Fig. 3C). Radial axons of the *Talpid3* mutant CA1, likely to be pyramidal neurons, appeared to be similar to control CA1. (Fig. 3D). This suggests that pyramidal neurons and connections, both in/out of the hippocampus, appeared to develop normally. However, the dentate gyrus appeared smaller and underdeveloped at this stage in the *Talpid3* mutant mice.

### Progenitor and mature neurons are mislocalized in the *Talpid3* mutant hippocampus

Neurogenesis is regulated by the sequential expression of transcription factors. In the developing and adult DG, proliferating cells are classified as type1, type 2 and type 3 based on the transcription factors they express. Type 1 cells are radial glial cells (neural stem cells-NSCs) and express Pax6, GFAP and Sox2. Type 2 cells express Tbr2 and are the transit amplifying cells or the intermediate progenitors and the Type3 cells are the committed neuroblasts that express NeuroD (31,32).

**Figure 4:**
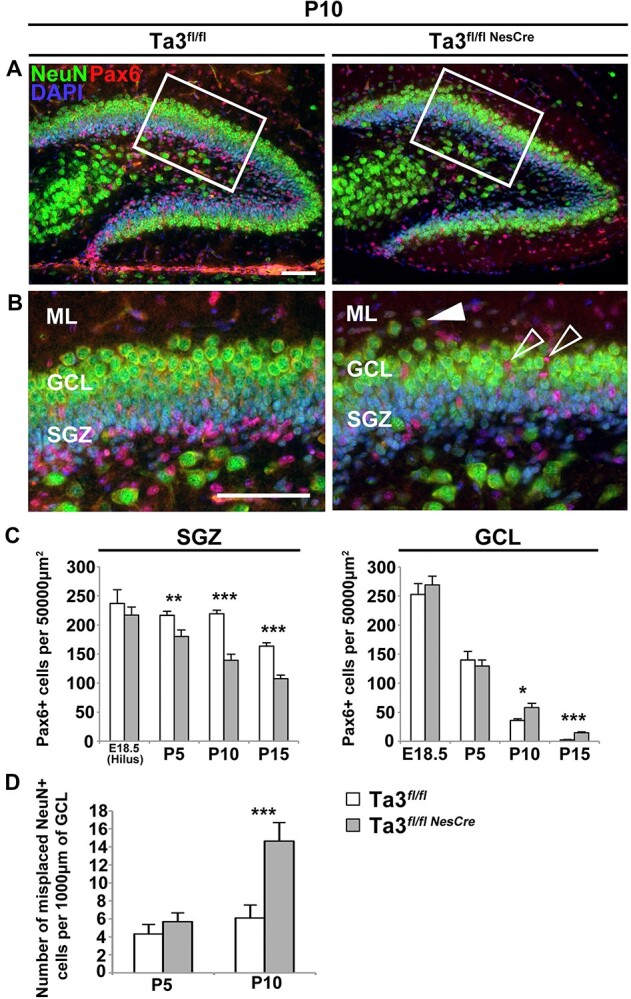
Mutant (Ta3^fl/flNesCre^) hippocampus has fewer SGZ progenitors, misplaced GCL progenitors and ectopic mature neurons. (**A**) P10 dentate gyrus immuno-stained for NeuN and Pax6. Box indicates region of higher magnification in (**B**). Mutants (Ta3^fl/flNesCre^) exhibit a loss of Pax6+ progenitors in the SGZ but an increase in the GCL (open arrows). Mutants (Ta3^fl/flNesCre^) also show ectopic NeuN^+^ neurons mislocalized outside of the GCL (Solid arrow). (**C**) Quantification of Pax6 progenitors in the SGZ and GCL. In the mutant (Ta3^fl/flNesCre^) SGZ progenitors are significantly reduced from P5 onwards. Progenitor numbers are significantly increased in the GCL of mutants (Ta3^fl/flNesCre^) from P10 onwards. (**D**) Quantification of NeuN^+^ neurons mislocalized outside the GCL. Mutants (Ta3^fl/flNesCre^) show significantly higher numbers of mislocalized NeuN^+^ neurons from P10 onwards. Scale bars: 75 μm (A, B). Error bars: (C, D) SEM (*n* = 3), ^*^P ≤ 0.05, ^**^P ≤ 0.01, ^***^P ≤ 0.001, Student’s *t*-test.

Progenitor and mature neurons of the hippocampus were identified by staining for Pax6 and NeuN respectively during the development of the dentate gyrus (E18.5, P5, P10 and P15) in the *Talpid3* mutant and control mice (Fig. 4). We found that the outer boundary of the GCL in the *Talpid3* mutant was less well defined when compared to the control mice at P10. At this stage, we also observed a 2.4 fold increase in the number of NeuN-positive cells mis-localized in the ML (Fig. 4A, B and D). *Talpid3* mutants also had a significantly fewer numbers of Pax6 positive progenitors in the developing SGZ (Fig. 4A–C), 37% and 34% at P10 and P15, respectively (Fig. 4C). The other unexpected finding was the incidence of ectopic Pax6-positive progenitors in the GCL of the *Talpid3* mutant (Fig. 4B and C). At younger stages between E18.5-P5 significant numbers of progenitors were present in the control GCL, reflecting the cell migration in the SPZ-to-hilar transition (30). However, this was not the case at later stages. Quantification of progenitor numbers from three control and three *Talpid3* mutant mice at P15, revealed that the *Talpid3* mutants had a 6.1 fold higher number of Pax6-positive progenitors in the GCL compared to their sibling controls (Fig. 4C).

### *The Talpid3* mutant hippocampus exhibits a loss of radial glial progenitors and intermediate progenitors

NSCs can exist in an activated state or inactivated state and the minichromosome maintenance protein 2 (MCM2) can be used to distinguish these two states. Ki67, PCNA (proliferating cell nuclear antigen), and MCM2 are endogenous markers that we used to label the status of NSCs. MCM2 is expressed during all phases of the cell cycle but is downregulated when cells exit the cell cycle into a quiescent state as for example it has been shown that quiescent intestinal stem cells downregulate MCM2 (33). The nuclear protein Ki67 is expressed through the cell cycle but not in the G0 or early G1 phase (34). Similar to Ki67, cells in late G1, S and G2-M phases can be identified by the expression of Proliferating cell nuclear antigen (PCNA). Both these markers are associated with actively proliferating cells. In contrast, expression of MCM2 identifies all activated cells, i.e. cells leaving G0 to go to G1 phase, which means it identifies cells with proliferative potential (35,36,37). The Mcm2/Ki67 ratio, can therefore be used to estimate the population of cells that are in early G_1_ (licensed to proliferate).

**Figure 5:**
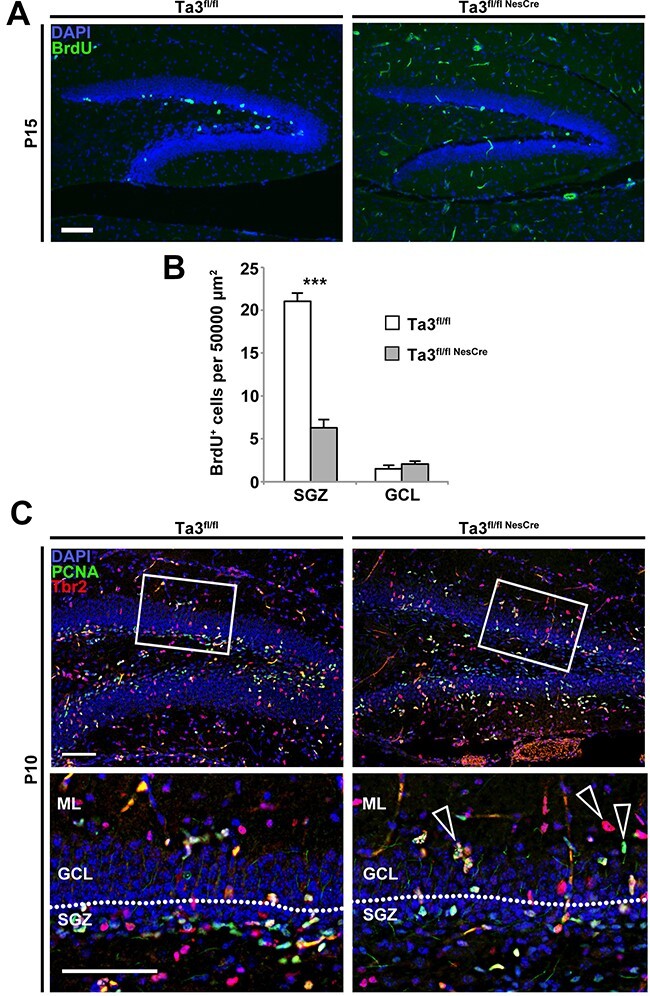
*Talpid3* mutant SGZ has fewer radial glia cells and intermediate progenitors. BrdU was administered to P15 mice 2 h before euthanasia. (**A**) Immunohistochemical identification of BrdU incorporation in the dentate gyrus. BrdU positive cells were easily identifiable in the subgranular zone (SGZ) but few were visible in the GCL. (**B**) Quantitation of BrdU+ cells demonstrated that mutants (Ta3^fl/flNesCre^) showed a significant reduction in the SGZ. (**C**) P10 mutant (Ta3^fl/flNesCre^) and control (Ta3^fl/fl^) dentate gyrus immuno-stained for PCNA and Tbr2. Mutant (Ta3^fl/flNesCre^) exhibit loss of progenitors in the SGZ and ectopic progenitors in the SGZ (arrows). Scale bars: 100 μm (A), 75 μm (C). Error bars: (B) SEM (*n* = 3), ^***^P ≤ 0.001, Student’s *t*-test.

To determine the number of cycling cells in the hippocampus, Bromodeoxyuridine (BrdU) was administered to P15 mice 2 h before euthanasia and stained for BrdU+ve nuclei. *Talpid3* mutants showed a 70% reduction in the number of cycling cells in the SGZ (Fig. 5A and B). At this stage, only a low number of BrdU-positive cells were identified in both control and mutant GCL and a significant difference was not observed (Fig. 5B).

Intermediate progenitors (type 2b) and proliferating cells were identified by labelling for Tbr2 and Proliferating cell nuclear antigen (PCNA), respectively in the P10 dentate gyrus (Fig. 5C). Proliferating cells which are PCNA-positive RGC (type-1, Tbr2 negative) or non-radial glial progenitors (type-2a, Tbr2 positive). The *Talpid3* mutant dentate gyrus showed a reduction in the numbers of glial progenitors and intermediate progenitors, which was particularly striking in the SGZ.

The reduction in the number of progenitors was confirmed by co-immuno-staining cells for the activated precursor marker MCM2 and for the proliferation marker Ki67 (Supplementary Material, Fig. S1A). At e18.5, quantification of the number of MCM2-positive or Ki67-positive cells in the dentate gyrus from three control and three *Talpid3* mutant mice showed no significant difference (Supplementary Material, Fig. S1B and C). Quantification in the SPZ (Supplementary Material, Fig. S1B and C) and in the primitive GCL or the developing hilus below (Supplementary Material, Fig. S1D and E) also did not show any statistical difference in the progenitor numbers. The similarity between control and *Talpid3* mutant dentate gyrus progenitors was confirmed by staining the E18.5 dentate gyrus for additional proliferation markers PCNA and PH3 (Supplementary Material, Fig. S1D). Consistent with previous observations, the density of cycling cells and their level of proliferation was very comparable. Closer examination of sections stained with Pax6 and NeuN also exhibited little difference between control and *Talpid3* mutant as progenitors were clearly present in the SPZ and GCL (Supplementary Material, Fig. S1E) at this stage. This suggests that, despite loss of primary cilia in the *Talpid3* mutant at E18.5, the defective proliferation in the hippocampus is evident only in the postnatal stages.

**Figure 6:**
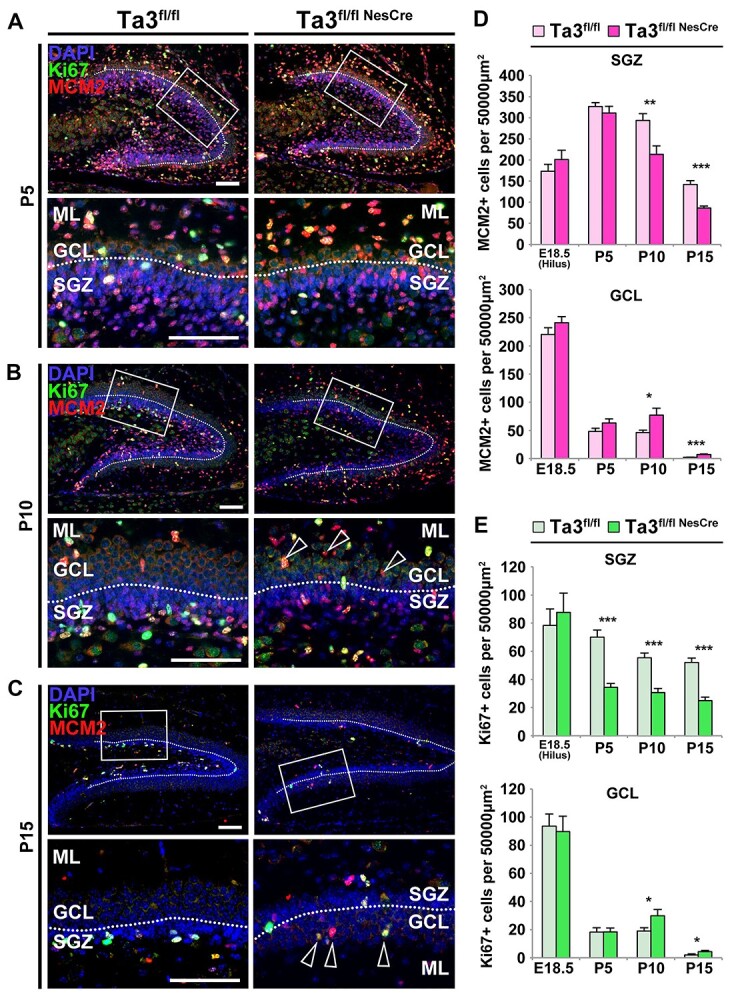
Loss of SGZ progenitors and occurrence of ectopic GCL progenitors in *Talpid3* mutant mice. Dentate gyrus immuno-stained for Ki67 and MCM2 at P5 (**A**), P10 (**B**) and P15 (**C**). Boxed areas indicate regions of higher magnification. Hollow arrows indicate ectopic progenitors in the mutant GCL. Note that in (C) area of higher magnification is on lower blade of DG resulting in reversed order of subgranular zone (SGZ), GCL and ML (ML). (**D**) Quantification of total MCM2+ progenitors. Mutant (Ta3^fl/flNesCre^) shows significant loss of MCM2+ cells in the SGZ and significant gain in the GCL from P10 onwards. (**E**) Quantification of total Ki67+ progenitors. Mutants (Ta3^fl/flNesCre^) have significantly less Ki67+ cells in the SGZ from P5 onwards and significant gain in the GCL from P10 onwards. Scale, 75 μm (A–C). Error bars: (D, E) SEM (*n* = 3), ^*^P ≤ 0.05, ^**^P ≤ 0.01, ^***^P ≤ 0.001.

We further quantified cells in the post-natal hippocampus of *Talpid3* mutant and control siblings which were immuno-stained for MCM2 and Ki67 at P5, P10 and P15 (Fig. 6A–E). *Talpid3* mutants exhibited a significant decrease (51%) in the total number of proliferating (Ki67 positive) cells in the P5 SGZ but no reduction was seen in the number of MCM2 positive progenitors when compared to control (Fig. 6A, D and E). The total number of proliferating cells was also significantly reduced (45%) in the SGZ of the *Talpid3* mutant at P10. In addition, although not significant, there appeared to be a reduction in the number of MCM2-positive progenitors (Fig. 6B, D and E). This difference in the SGZ became more apparent at P15, with *Talpid3* mutants exhibiting a 63% reduction in MCM2-positive progenitors and 71% reduction in Ki67-positive proliferating cells compared to the control siblings (Fig. 6C, D and E).

To better assess the progenitor population in the *Talpid3* hippocampus, the number of cells which were single or double-labelled for MCM2 and Ki67 were quantified at P5, 10 and 15. Compared to control siblings, *Talpid3* mutants were found to have a greater proportion of non-dividing but activated SGZ progenitors (MCM2-positive, Ki67-negative) (Supplementary Material, Fig. S2A and B). This meant that in addition to having fewer MCM2-positive progenitors in the SGZ, fewer of these were also less able to actively proliferate leading to a depleted progenitor pool.

GFAP is used as a marker of radial and non-radial glial progenitors (type 1 and 2a), but it is also present in astrocytes of the dentate gyrus. By double labelling for GFAP and Ki67 it is possible to preferentially identify the proliferating glial progenitor population (Supplementary Material, Fig. S2C and D). Quantification of the proportion of Ki67-positive progenitors that were GFAP positive or GFAP negative demonstrated that there was no statistical difference between the proportions of glial and non-glial progenitors in the P15 SGZ when comparing control and *Talpid3* mutant (Supplementary Material, Fig. S2E). Given that the total number of Ki67-positive cells was reduced, we concluded that there is a decline in the number of both glial progenitors and intermediate progenitors in the SGZ. This is the likely to be the cause of the hypoplasia seen in the GCL at later stages in the *Talpid3* mutant mice.

Despite the progressive depletion of progenitors seen in the mutant SGZ, the total number of Ki67-positive cells appeared to show a slight increase from P10 onwards (Supplementary Material, Fig. S2D and E). Although these data were not significantly different, they follow the same trend seen with the numbers of Pax6-positive cells in the *Talpid3* mutant GCL at this age (Fig. 4C). By P15 the total numbers of MCM2-positive and Ki67-positive cells in the control and *Talpid3* mutant GCL had reduced, however, the *Talpid3* mutants still appeared to show significantly more MCM2-positive cells compared to the control (Supplementary Material, Fig. S2A and B). Analysis of single- and double-positive cells showed that the GCL in the P15 control had a greater proportion of proliferating cells which are MCM2-negative (Ki67-positive, MCM2-negative) compared to P5 and P10 GCL (Supplementary Material, Fig. S2B). This marks a shift in the type of proliferating cell present in the GCL and indicates the completion of the SPZ-to-hilar transition. In addition, the *Talpid3* mutant GCL at P15 had 25% higher number of proliferating cells which are MCM2-positive (Ki67-positive, MCM2-positive) compared to the control GCL (Supplementary Material, Fig. S2B). Interestingly, the number of ectopic cells seen in the P15 *Talpid3* mutant GCL more closely resembled those seen in the P15 SGZ of control sibling (Supplementary Material, Fig. S2A and B). Furthermore, a higher percentage of the proliferating cells in the *Talpid3* mutant GCL were GFAP-positive, which suggests that the ectopic progenitors found in the mutant GCL are mis-localized glial-progenitors (Supplementary Material, Fig. S2E and F).

### Mislocalized progenitors correlate with poorly formed cellular scaffolds

**Figure 7:**
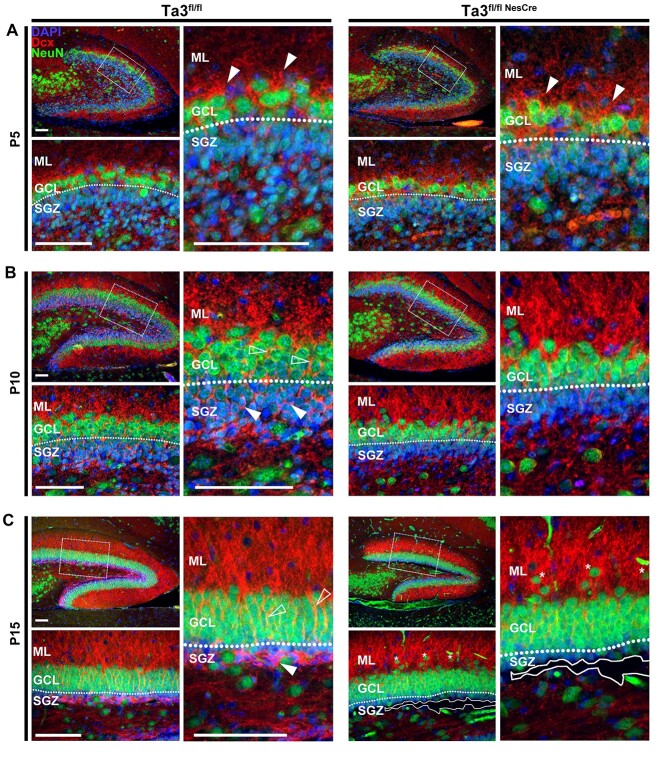
*Talpid3* mutant dentate gyrus exhibits loss of Dcx in the SGZ and GCL. Dentate gyrus immuno-stained for Dcx and NeuN. (**A**) In the P5 dentate gyrus, the majority of Dcx staining is observed at the boundary of the GCL and ML (ML) (solid arrows). Control (Ta3^fl/fl^) and mutant (Ta3^fl/flNesCre^) show comparable levels of Dcx. (**B**) The P10 control (Ta3^fl/fl^) dentate gyrus has increased levels of Dcx with fibres found in the SGZ (solid arrows) and GCL (open arrows). This increase is far less evident in mutant (Ta3^fl/flNesCre^) SGZ and GCL. (**C**) P15 control (Ta3^fl/fl^) dentate gyrus has thick band of Dcx in SGZ (solid arrow) with fibres stretching throughout the GCL (hollow arrows). Mutant DG (Ta3^fl/flNesCre^) exhibits a striking loss of Dcx in both regions. Loss of tissue integrity is indicated by solid line. Asterisks indicate mature neurons mislocalized in the ML (ML). Scale bar: 75 μm (A–C).

Doublecortin (Dcx) is a cytoskeleton component closely associated with neurogenesis and migration (38,39). In order to explain the presence of misplaced progenitors and mature neurons in the *Talpid3* mutant dentate gyrus, the distribution of Dcx in the dentate gyrus was analyzed between E18.5 and P15 (Fig. 7A–C). The expression of Dcx in the control dentate gyrus at e18.5 was very low and was hard to detect immunohistochemically. The levels of Dcx increased at P5, with the strongest expression at the boundary of the GCL and ML (Fig. 7A). At this stage, Dcx expression was similar between the control and *Talpid3* mutant sibling mice. However, in the P10 dentate gyrus of control sibling mice, there was an increase in the level of Dcx in the SGZ (Fig. 7B). Dcx-positive fibres seen spanning the GCL towards the ML in the control mice was not observed in the *Talpid3* mutant SGZ or GCL.

The levels of Dcx expression in SGZ of the control mice increased further and at P15 was evident as a thick band labelling the SGZ (Fig. 7C). The Dcx positive fibres stretching through the GCL were also more obvious and many could be traced from SGZ through to the ML. In contrast, the *Talpid3* mutant showed a striking absence of the thick Dcx band in the P15 SGZ and this correlated with the loss of tissue integrity (indicated by solid line, Fig. 7C). Dcx fibres stretching through the *Talpid3* mutant GCL at P15 were also far less distinct. Although the distribution of Dcx in the *Talpid3* mutant demonstrated a prominent phenotype, it seemed to correlate more with the loss of progenitors in the SGZ. Given the ectopic progenitors seen in the *Talpid3* mutant GCL, it would seem reasonable to expect increased Dcx positive fibres, however, this was not observed at the later stages.

**Figure 8:**
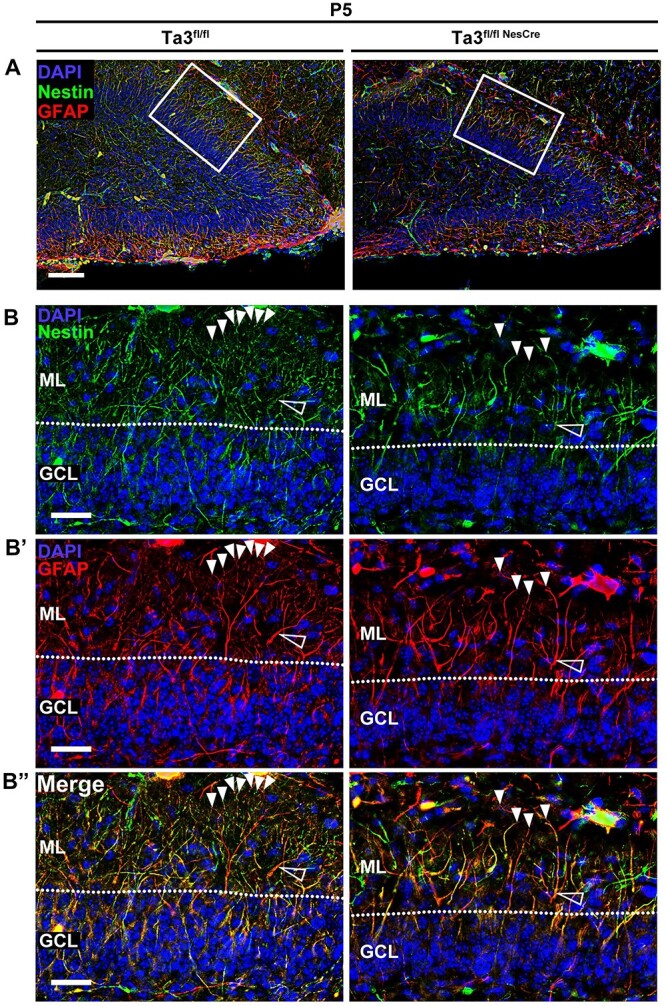
The glial scaffold is defective in *Talpid3* mutant dentate gyrus. P5 dentate gyrus immuno-stained for nestin and GFAP. (**A**) Image of dentate gyrus with boxed region indicating region of higher magnification. (**B**) nestin expression, (**B**′) GFAP expression, (**B**″) merge. Loss of fine widely branched fibres (solid arrows) and primary fibres which appear thickened (hollow arrow) is seen in Mutant (Ta3^fl/flNesCre^). Scale bars: 75 μm (A), 25 μm (B).

Many of the cell movements in the developing hippocampus are thought to be directed by glial-guided migration along radial fibres. The glial scaffold was assessed at P5 by co-labelling for GFAP and nestin where double-positive cells represent radial glial-progenitors (type 1 and type 2a) (Fig. 8A) (40). In the control dentate gyrus, both GFAP-positive and nestin positive fibres extended radially through the GCL and branch extensively in the ML. We observed two types of fibres; thicker primary fibres with simple radial organization, which then bifurcated into thin diffuse fibres with widespread branching. Upon closer examination, all visible fibres shared some co-localization of both GFAP and nestin (Fig. 8B). In the thicker primary fibres, GFAP had a more continuous distribution, whereas nestin was seen often intermittently along the length of the fibre. This high level of co-localization is a result of the large number of radial glial progenitors present at this age. The *Talpid3* mutant dentate gyrus showed a dramatic loss of both types of fibre (Fig. 8B″). Most obvious was the loss of fine branches in the ML. The reduction in the numbers of primary fibres is likely to represent a loss of RGC progenitors. In addition, the loss of fine branching suggests a defect in the glial cell morphology.

It is likely that the glial defect contributes to the ectopic progenitors seen in the *Talpid3* mutant GCL. To test this, sections of brain were co-immunostained for GFAP and the progenitor marker Pax6. At E18.5, the control sibling mice exhibited a dense network of GFAP-positive fibres in the SPZ with a small number of fibres present in both the hilus and presumptive GCL (Supplementary Material, Fig. S3A and B). In comparison, the *Talpid3* mutant hippocampus showed a dense GFAP network in the SPZ in which there appeared to be a very small reduction in the density of fibres. The numbers of Pax6 positive progenitors were comparable between control and mutant at E18.5 (Supplementary Material, Fig. S3A and B) as observed earlier.

**Figure 9:**
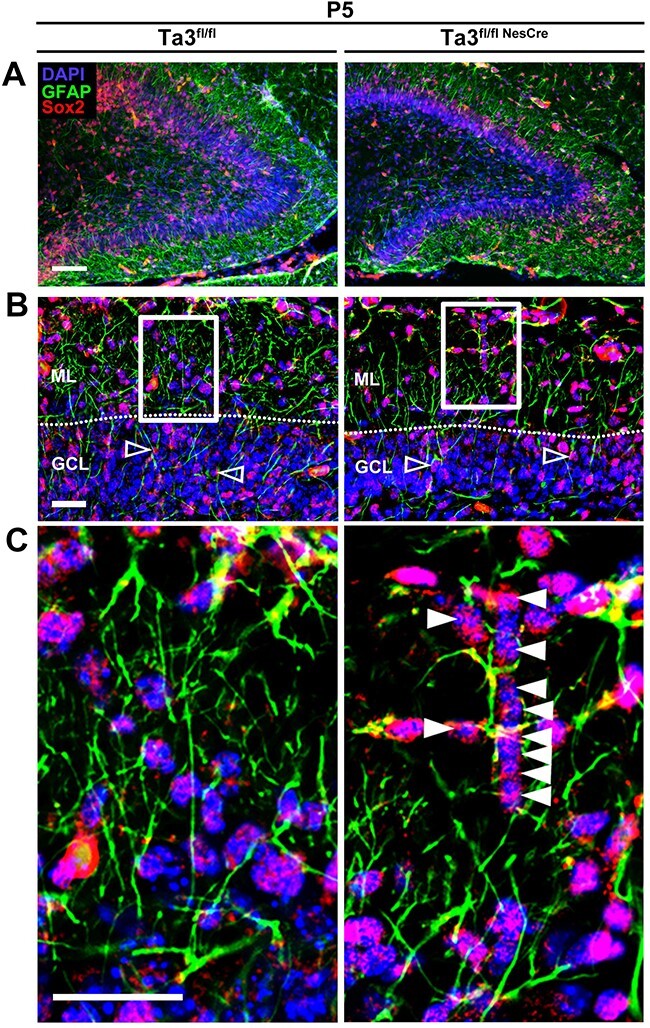
*Talpid3* mutant glia scaffold is abnormal and insufficient for progenitor migration. (**A**–**C**) P5 dentate gyrus immuno-stained for GFAP and Sox2. Loss of GFAP fibres in the ML (ML) and radial fibres stretching through the GCL is seen in the mutant (Ta3^fl/flNesCre^). Sox2+ progenitors are evenly distributed across the glial scaffold in the control (Ta3^fl/flNesCre^). In the mutant (Ta3^fl/flNesCre^) many progenitors are found clustered on a single fibre (Solid arrow). Box indicates region of higher magnification. Scale bars: 75 μm (A), 25 μm (B, C).

The *Talpid3* mutant dentate gyrus at P5 exhibited a striking reduction in the occurrence of glial fibres and extent of their branching but showed no difference in the number of progenitors present in the GCL (Supplementary Material, Fig. S3B). Type 1 and type-2b progenitors were identified by immunostaining for Sox2 in addition to GFAP (Fig. 9A–C). In the *Talpid3* mutant, many of the progenitors migrating through the GCL were not obviously associated with glial fibres and in addition several glial fibres appeared overloaded with progenitors on a single fibre (Fig. 9C). This is in contrast to the control mice, which had similar numbers of progenitors in the dentate gyrus but were better distributed through the glial scaffold. It is possible that the disrupted scaffold in the *Talpid3* mutant dentate gyrus limits access to glial fibres or restricts their pathway to the SGZ. This in-turn could delay or inhibit their SPZ-to-hilar transition resulting in ectopic progenitors evident in the GCL from P10.

Reelin, an extracellular protein expressed by Cajal–Retzius cells in the ML plays an important role in the migration of hippocampal neurons and their dendritic branching (41). Reelin has been shown to be important in the movement of progenitors away from the SPZ towards the SGZ (41). It has also been demonstrated that Reelin is required for the correct formation of the RGC scaffold (38,42) and migration of post-mitotic granule neurons born in the SGZ (43–45). In the control P5 dentate gyrus, Reelin producing cells were clearly seen at the outer edge of the ML (Supplementary Material, Fig. S4). In the *Talpid3* mutant dentate gyrus Reelin producing cells were also present at the edge of the ML. However, there appeared to be a slight reduction in the number of Reelin-positive cells, which was most evident in the posterior region above the dorsal blade of the dentate gyrus (Supplementary Material, Fig. S4). This defect may be a contributing factor to the disrupted migration seen by granule progenitors or post-mitotic granule neurons.

### *Talpid3* and cell migration

Many of the phenotypes that we observed in the *Talpid3* mutant hippocampus are due to the complex interactions between the migrating cells and the glial scaffold. We took an *in vitro* approach to begin to address the questions arising from our findings on the *Talpid3* mutant mouse hippocampal phenotype by using hippocampal neurosphere cultures (Supplementary Material, Fig. S5). Hippocampal neurosphere lines were generated from postnatal P5 mice obtained by breeding *Talpid3fl/fl;UbcCreER^T2^* males to *Talpid3^fl/fl^* females as described in Materials and Methods. The expression of Cre in the *UBcCreER^T2^* mouse driver line can be induced by administering 4-hydroxytamoxifen (4-OHT). Each neurosphere line was created from the hippocampus of an individual mouse. In total, 13 independent hippocampal neurosphere lines each of *Talpid3fl/fl;UbcCreER^T2^* and *Talpid3^fl/fl^* genotype were generated. Neurosphere lines were expanded, disassociated (46) and frozen. This allowed all experiments to be performed using cells of identical passage number from frozen stocks. In total, three independent neurosphere lines of genotype *Talpid3fl/fl;UbcCreER^T2^* were selected for experimental analysis (designated ‘#1, 2, 3’) (Supplementary Material, Fig. S5). In addition, neurosphere lines from *Talpid3^fl/fl^* mice were also used as controls in the experiments.

We found that treatment of neurospheres with two doses of 4-OHT, one immediately after disassociation (0 day) and one 3 days later (3 days) led to robust recombination of *Talpid3*. The DNA from *Talpid3fl/fl;UbcCreER^T2^* neurospheres treated with and without 4-OHT for 7 days growth were analyzed by PCR for the recombination event (Supplementary Material, Fig. S6) showed a consistently high level of recombination caused by 4-OHT administration. Using this approach, we studied the consequences of *Talpid3* loss from postnatal hippocampal progenitors using the experimental plan illustrated in Supplementary Material, Figure S5.

### Neurospheres with a targeted deletion of *Talpid3* exons 11 and 12 formed smaller colonies

**Figure 10:**
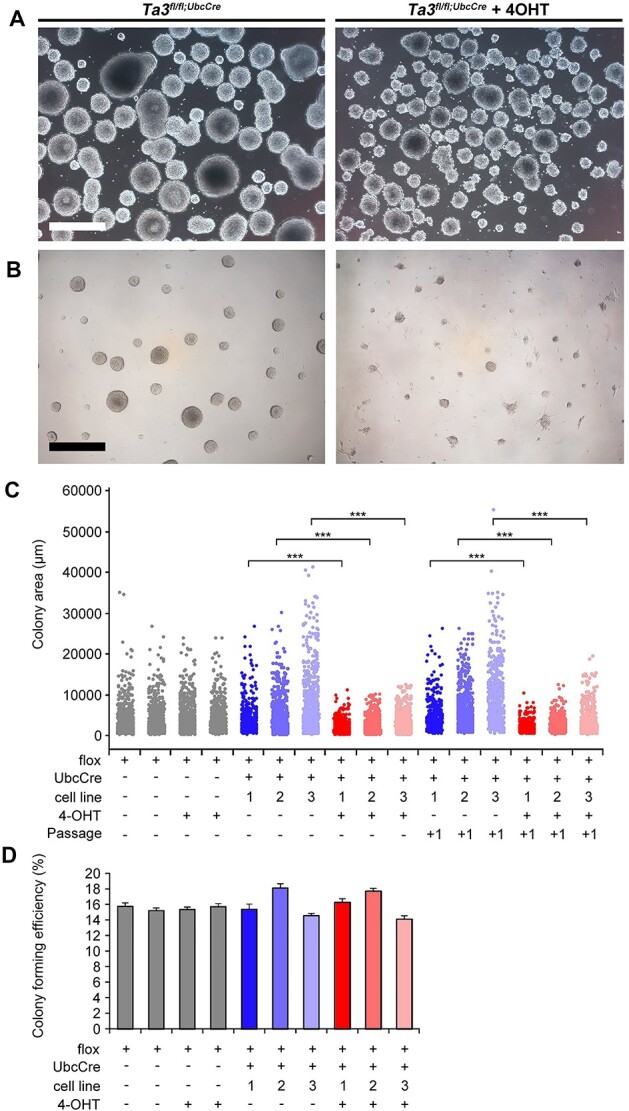
Loss of *Talpid3* leads to smaller colonies but with comparable colony forming efficiency. (**A**) Representative images of control and tamoxifen (4-OHT) administered (Ta3^fl/fl^UbcCre^T2^) neurospheres after 7 days growth. 4-OHT treated *Ta3^fl/fl^UbcCre^T2^* neurospheres are smaller. (**B**) Control and tamoxifen (4-OHT) administered *Ta3^fl/fl^UbcCre^T2^* grown as adherent colonies. Colonies were smaller in 4-OHT treated cultures. (**C**) Dot plot showing individual colony area measurements. Grey Dots: Control *Talpid3^fl/fl^* cells grown with and without 4-OHT. Blue dots: Control *Talpid3fl/fl;UbcCreER^T2^* cells without 4-OHT administration. Red dots: *Talpid3fl/fl;UbcCreER^T2^* cells with 4-OHT administration. *Talpid3fl/fl;UbcCreER^T2^* 4-OHT treated neurospheres consistently showed significant reduction in colony area. (**D**) Quantification of colony forming efficiency of *Ta3^fl/fl^UbcCre^T2^* neurospheres with and without 4-OHT treatment. Scale bar: 500 μm (A, B). Statistical comparisons were made between the same neurosphere lines. Error bars: (C) dot plot: >500 colony measurements per condition. ^***^P < 0.001, two-tailed Mann–Whitney test. (D) SEM (*n* = 12), no significant differences (two-tailed Student’s *t*-test).

The *Talpid3* mutant hippocampus exhibited a loss of proliferating progenitors. To assess the consequences of the deletion in *Talpid3* in the neurosphere lines we first monitored the effect on the growth of the neurospheres. *Talpid3^fl/fl^UbcCreER^T2^* neurospheres with and without with 4-OHT treatment were cultured for 7 days. Loss of *Talpid3* resulted in smaller neurospheres (Fig. 10A).

One problem encountered with neurosphere culture is that free-floating cultures often fuse resulting in size heterogeneity. To limit this effect and study neurosphere growth, disassociated neurosphere cells were plated in 96-well tissue culture plates at low density. Cells proliferated and grew as a round adherent colony derived from a single cell (Fig. 10B). Measurement of the area of the colony from multiple wells showed that loss of functional *Talpid3* resulted in significant reduction in median colony area in 4-OHT treated *Talpid3^fl/fl^UbcCreER^T2^* derived colonies as compared to untreated *Talpid3^fl/fl^*UbcCre cells (Fig. 10C). An important control included the use of *Talpid3^fl/fl^* cells with and without 4-OHT administration, which showed no difference in colony size. This confirms that the defect seen was due to loss of functional *Talpid3* rather than any adverse effect of 4-OHT. To assess the long term effect of the deletion in *Talpid3*, neurospheres cultured with and without 4-OHT administration were disassociated after 7 days and plated on 96-well tissue cultures plates (named ‘passage +1’) (Fig. 10C). These cells did not have any further addition of 4-OHT, yet colonies consistently showed a significant reduction in median colony size when compared with treated and untreated controls. This suggests an intrinsic and permanent defect caused by the deletion in *Talpid3*.

### Inducible deletion of exons 11–12 of *Talpid3* in differentiated cells leads to loss of cilia and a disrupted actin cytoskeleton

We assessed the consequence of deletion of exons 11 and 12 in *Talpid3* on primary cilia in differentiated cells derived from neurospheres by immunostaining for ACIII and acetylated α-tubulin. Cells with a deletion in *Talpid3* had considerably fewer primary cilia (Supplementary Material, Fig. S7B). Primary cilia were detected on 61% of cells in neurospheres that had a functional *Talpid3*, whereas only 9% *Talpid3* mutant neurosphere cells possessed a primary cilia (Supplementary Material, Fig. S7C). This confirmed that deletion of exons 11 and 12 of *Talpid3* had occurred in a significant number of cells which was sufficient to cause loss of primary cilia.

Previous reports have suggested that loss of *Talpid3* leads to defects in the actin cytoskeleton (16,17). Staining with fluorescently labelled phalloidin was used to detect stabilized F-actin in differentiated cells generated from neurospheres as described in Materials and Methods (Supplementary Material, Fig. S8A), which showed a reduction in the number of stress fibres in cells with a deletion in *Talpid3* (Supplementary Material, Fig. S8B). Importantly, however, increased levels of actin in the ruffled membrane or filopodia were not observed in these cells.

### Deletion of exons 11 and 12 of *Talpid3* affects migration of cells from neurospheres

**Figure 11:**
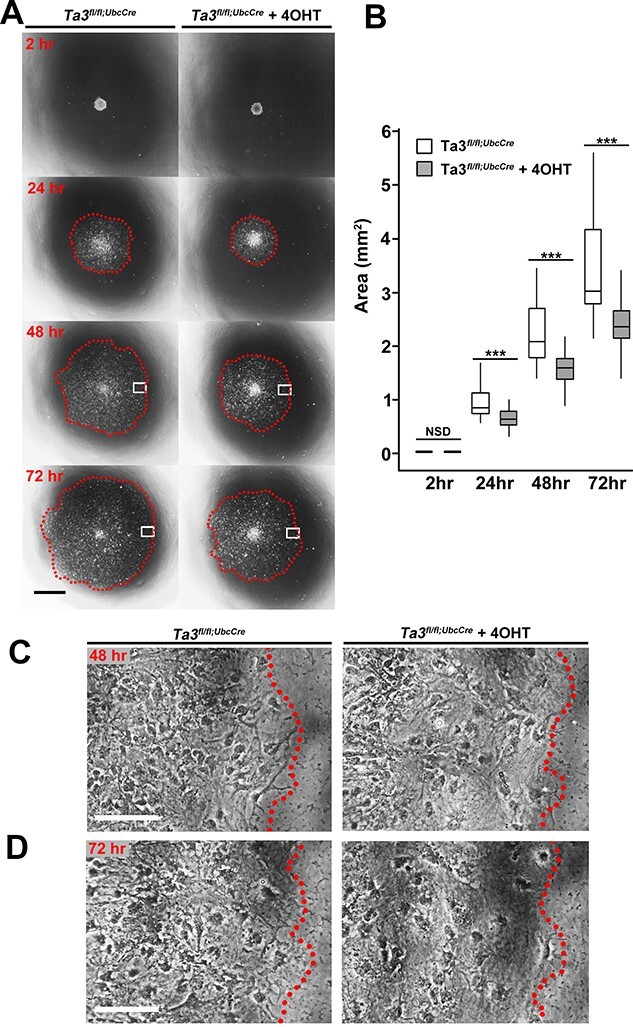
Loss of *Talpid3* affects migration in the neurosphere migration assay. (**A**) Representative images of neurosphere migration assay where 7 day Ta3^fl/fl^UbcCre^T2^ neurospheres (with or without tamoxifen administration) are plated on matrigel and their migration monitored over 72 h. Dotted line indicates maximum extent of migration at 72 h. Boxed region indicates location of higher magnification shown in (**C**, **D**). (**B**) Quantification of migration areas shows that loss of *Talpid3* results in a reduced migration distance 24, 48 and 72 h after plating. After (C) 48 h and (D) 72 h, cells at the outer edge appear more diffuse following loss of *Talpid3*. Scale bar: 500 μm (A), 100 μm (C, D). Error bars, box plot (control; *n* = 36, 4OHT; *n* = 35),^***^P < 0.001 (one-tailed Mann–Whitney test).

One commonly used approach to monitor the capacity of neural cells to migrate is the neurosphere migration assay (47,48,49,50). In this paradigm, neurospheres of equal size are transferred to individual wells coated with Matrigel^®^ containing differentiation media. Migration of cells away from the neurosphere was monitored over the course of 72 h to provide a quantitative readout of neural migration (Fig 11A). Due to the nature of this assay, it is a useful system to monitor the fundamental ability of cells to migrate, rather than in response to signalling gradients.

Outgrowths from both 4-OHT treated and untreated 7-day *Talpid3^fl/fl^UbcCre* neurospheres from three independent neurosphere lines were measured after 2, 24, 48 and 72 h of culture. At all the time points, OHT-treated cells had migrated significantly less distance than the untreated controls (Fig. 11B). In addition to the smaller radial area, the leading edge of the treated cultures had a lower density of cells after 48 h and this was maintained after 72 h (Fig. 11C and D).

## Discussion

We have previously shown that conditional deletion of the exons 11 and 12 of *Talpid3* in the CNS leads to very severe cerebellar defects reminiscent of JS (29). Here we show that in addition to the cerebellar defects, the *Talpid3* mutant mice exhibit prominent defects in the dentate gyrus. The *Talpid 3* mutant hippocampus shows a reduction in the numbers of progenitors in the SGZ and ectopic progenitors in the GCL, which correlates with aberrant formation of the underlying glial scaffold. Here we show that defects occur in the SPZ-to-hilar transition in the hippocampus in addition to the proliferative defects described in other cilia deficient mutant mice.

The defective proliferation and aberrant progenitor localization in the *Talpid3* mutant dentate gyrus is somewhat similar to that seen in the hippocampus of *B9d2^fl/fl^NesCre* mice (27). Breunig *et al*. (27) found that the dentate gyrus of the *B9d2* mutant mice at P0 was smaller with reduced proliferation. The *Talpid3* mutant dentate gyrus appears to be normal at e18.5, and the first signs of defects were evident at P0 similar to the *B9d2* mutant. It appears that the earliest stage of dentate gyrus development requiring *Talpid3* is P0. In contrast, loss of *Kif3*a using the hGFAP-Cre deleter (*Kif3*a*^fl/fl^* hGFAPCre) resulted in a smaller dentate gyrus with reduced proliferation at e18.5 (26).

Both *NesCre* and *hGFAP-Cre* deleters have been shown to cause widespread recombination from mid-gestation onwards (28,51,52). It is likely that the defect in *Talpid3^fl/fl^NesCre* dentate gyrus was not observed at an earlier stage because of the slightly different times of onset of recombination mediated by the *hGFAP-Cre* and *NesCre* deleter strains. However, it is also tempting to speculate that this difference in phenotype may be due to the differing roles of *Talpid3* and *Kif3a*. It is plausible that the earlier phenotype seen in *Kif3a^fl/fl^ hGFAPCre* dentate gyrus compared to the *Talpid3^fl/fl^NesCre* and *B9d2^fl/fl^NesCre* dentate gyrus may be due to a cilia-independent role of *Kifa3a* in the processing of Gli3.

The onset of defects in the dentate gyrus in the *Kif3a^fl/fl^hGFAPCre* occurs earlier than in the *Talpid3^fl/fl^NesCre* and *B9d2^fl/fl^NesCre* mice but all three mutants exhibit defective proliferation in the dentate gyrus (26,27). Han *et al*. extended this further and showed that there was a reduction in proliferation in the dentate gyrus of a hypomorphic *IFT8*8 mutant (*IFT88orpk/orpk*) and *Ftm* (*Rpgrip1l*)*^−/−^* mice.

In the *Talpid3* mutant dentate gyrus, there is loss of RGC (type 1), non-radial glial progenitors (type 2a) and intermediate progenitors (type 2b) resulting in a depletion of the progenitor pool similar to that seen in the *B9d2^fl/fl^NesCre* mice that showed a loss of slow cycling progenitors and greater exit from the cell cycle (27). In the case of both *B9d2* and *Kif3a* mutant mice, the defect in proliferation was attributed to loss of Shh signalling. The hippocampus in the*Talpid3* mutant mice lack cilia resulting in a loss of Shh signalling. This is consistent with a loss of proliferation seen in *Smo^fl/-^NesCre* dentate gyrus (25). However, the phenotype in *Smo^fl/-^Ne*sCre dentate gyrus was more prominent with increased hypoplasia in the GCL. An even more severe phenotype was seen in the *Gli3^fl/fl^Emx1Cre* mice, in which the initial blade of the dentate gyrus failed to form by E18.5 (52).

The *Talpid3* mutant dentate gyrus had ectopic progenitors in the GCL, which is also seen in the dentate gyri of *B9d2^fl/fl^Nes*Cre and *Kif3a^fl/fl^hGFAPCre* (26,27). Here, for the first time, we draw a causal link between the two phenotypes and suggest that progenitors are mis-localized as a direct result of the defective glial scaffold. In addition, in the *Talpid3* mutant hippocampus, fewer progenitors were associated with radial fibres and those that were associated appeared to be overloaded. These observations suggest that the glial scaffold is insufficiently developed in the *Talpid3* mutant hippocampus and is inadequate for SPZ-to-hilar migration. It is also important to note that between the ages of P10 and P15 the *Talpid3* mutant showed a reduction in the number of ectopic progenitors in the GCL indicating that there may be a delayed or ineffective migration rather than a complete block.

We suggest a link between the glial scaffold abnormalities and defective progenitor migration in the *Talpid3* mutant mice; however, the exact contribution of radial glia in progenitor migration is still unclear. To address this, studies specifically targeting the glial scaffold in precise time windows during development are needed to explicitly show the role and requirement of radial glia for the SPZ-to-hilar transition. It is also unclear whether loss of *Talpid3* affects the glial branching directly or whether there is simply a reduction in the number of glial progenitors. Further studies looking at glial behaviour *in vitro* will help determine whether *Talpid3* is directly required for glial scaffold formation. The severe hydrocephaly and ataxia seen in *Talpid3* mutant mice precluded the study of the dentate gyrus at later stages using the *NesCre* deleter strain.

Reelin is a secreted protein, which is well known to influence the migration of neurons in the hippocampus. *Talpid3* mutant dentate gyrus showed a subtle reduction in the number of Reelin-positive cells in the ML. Mice with constitutive loss of Reelin, the so-called ‘Reeler’ mice, have a disruption of hippocampus with aberrant radial glial scaffold (38,43) and failure of the SPZ-to-hilar transition (42). Loss of Reelin signalling has also been shown to cause ectopic localization of post-mitotic granule neurons both in the hilus and ML (44,45). Reeler mutant mice also have a thinner entorhinal performant pathway termination zone in the ML (44). Studies of other primary cilia mutants have described relatively normal distribution of Reelin-positive cells in the marginal zone of the cortex of *Kif3a^fl/fl;hGFAPCre^* (53), *Arl13b^hnn/hnn^* (54) and hypomorphic *Ift88^cbs/cbs^* (55) mice. The wide ranging defects seen in mutants with complete loss of Reelin makes it difficult to interpret the effects of the subtle reduction in Reelin-positive cells in the *Talpid3* mutant hippocampus.

The *Talpid3* mutant dentate gyrus is characterized by the presence of ectopic post-mitotic granule neurons in the ML. Disc1, a protein known to interact with the basal body, has been shown to be required for primary cilia formation in cultured cells (56). Loss of DISC1 resulted in aberrant placement of granule neurons in the adult hippocampus similar to that seen in *Talpid3* mutant mice (57,58).

Neurospheres generated from the *Talpid3^fl/fl^UbcCreER^T2^* mice in which *Talpid3* could be deleted in an inducible manner by addition of 4-OHT allowed us to dissect the role of *Talpid3,* if any, on growth of neurospheres and migration of cells. Neurospheres in which *Talpid3* was deleted, lacked primary cilia and formed smaller colonies. In addition, they had a disrupted actin cytoskeleton and the cells showed a marked decrease their ability to migrate. The reduction in progenitor proliferation is consistent with the loss of dividing progenitors seen in the *Talpid3* mutant hippocampus. It is likely that the smaller colony size seen *in vitro* and fewer hippocampal progenitors seen *in vivo* are regulated by the same mechanism.

To date, there have been a few studies looking at the effects of loss of primary cilia in hippocampal cultures. Breunig and colleagues (27) showed that, in slice cultures, loss of primary cilia rendered hippocampal progenitors unable to respond to exogenous Shh. In neurospheres derived from the perinatal SVZ, cooperation between Shh and EGF signalling was shown to regulate their formation and growth (59). Using culture conditions similar to the current study, the Hh inhibitor, cyclopamine, was able to reduce neurosphere proliferation indicating the occurrence of autocrine or paracrine Hh signalling within neurospheres. Given the known roles Shh in the hippocampus *in vivo* (25–27), this signalling pathway is a prime candidate influencing the growth of hippocampal neurospheres following loss of *Talpid3*.

Microtubules did not appear to be affected in the *Talpid3* mutant neurospheres but had fewer F-actin stress fibres compared to controls. This is consistent with cytoskeletal phenotypes seen in other *Talpid3* deficient cell types including *talpid3* chick neural tube, cultured *talpid3* chick limb cells (16) and *Talpid3^flfl;PrrxCre^* mouse limb fibroblasts (17). Cells lacking IFT88 and basal body proteins BBS4, BBS6 and Mks3 have also been shown to have a disrupted actin cytoskeleton (61-63-56). The extent to which the disruption of actin cytoskeleton contributes to the hippocampal phenotype is unclear at present.

Cells from hippocampal neurospheres with a deletion in *Talpid3* exhibited retarded migration. This suggests that there are defects in cell migration, which are independent of the glial scaffold since fewer cells were seen at the leading edge. Migration differences in this assay are likely to be the result of intrinsic defects in the ability of cells to move, rather than their ability to orientate themselves towards a morphogen. Our experimental data suggest that both loss of some intrinsic property of migrating cells and the defective underlying glial scaffold contribute towards the *in vivo* migratory defect and failure of SPZ-to-hilar transition.

Fibroblasts lacking *Talpid3* have been described as having less directionality (17) in the scratch assay but we did observe any lack of directionality in cells migrating from the *Talpid3* mutant neurospheres. Scratch assays have been used to study the role of cilial components in different cell types including endothelial cells and mouse embryo fibroblasts (MEFs) with hypomorphic *IFT88* (60,63) and kidney cells lacking *BBS4* or *BBS6* (61,62). In all these cases, the cells lacked cilia and exhibited a reduced ability to migrate. MEFs have been shown to require primary cilia to transduce PDGFα signalling required for directional cell movement (64). It has been further shown that loss of IFT88 influences PDGFα signalling which has a resultant effect on MEK1/2—Erk1/2 pathway, ultimately affecting actin distribution at the lamellipodia of migrating fibroblasts (63). To what extent PDGFα influences cell migration in the neurosphere assay is not known but it has been shown that cells isolated from the hippocampus express components of the PDGFα signalling pathway (65).

In conclusion we have shown that the *Talpid3* mutant mice have defects in the hippocampus, which exhibits reduced proliferation and ectopic progenitor cells. This may have a bearing in the learning and cognitive defects in JS patients. The *Talpid3* mutant mice is an useful model to further unravel the molecular and cellular mechanisms underlying JS.

## Materials and Methods

### Mouse strains

Mice carrying the floxed *Talpid3* (*2700049A03Rik*) allele (*Talpid3^fl/fl^*) were generated as described previously (17) and were maintained as a homozygous line (*Talpid3^fl/fl^*) on an inbred C57/Bl6 background. The Cre deleter mouse strains (1) *B6.Cg-Tg(Nes-Cre)1kln/J* (*NesCre*) (28) and (2) *B6.Cg-Tg(Ubc-cre.EsR1)1Ejb/J* (UbcCreER^T2^) (66,67) were obtained from The Jackson Laboratory and maintained as hemizygous lines on an inbred C57/Bl6 background.

### Colony management

Mice were maintained on a 12 h light/dark cycle with access to food and water *ad libitum*. All procedures were approved by the University of Bath Ethical Review Board and were conducted under HO animal procedures project (VS) and personal licences (AB and VS) in accordance with UK Home Office guidelines and the UK Animals (Scientific Procedures) Act, 1986.

### Breeding to generate experimental mice

*Talpid3^fl/fl^* mice were bred to *NesCre* mice to produce *Talpid3^fl/wt;NesCre^* stud males. To obtain experimental embryos/mice, *Talpid3^fl/wt;NesCre^* stud males were crossed to *Talpid3^fl/fl^* female mice to generate *Talpid3 ^fl/fl;NesCre^* mice that are referred to as *Talpid3* mutant and *Talpid3^fl/fl^* (control) mice.

### Genotyping

Mice were genotyped using DNA isolated from tail biopsies or ear punches Genotyping was carried out by PCR using GoTaq^®^ Flexi DNA Polymerase (Promega, UK), according to manufacturer’s instructions. Primer sequences used for genotyping of the different mouse strains are shown in Supplementary Material, Table S1.

### Dissection and processing of tissues

Brains were dissected from postnatal mice euthanized by intraperitoneal injection of sodium pentobarbitone solution (200 mg/kg) (Euthatal; Merial Animal Health Ltd, UK). Dissected brains were rinsed in ice cold phosphate buffered saline (PBS), where necessary they were bisected in sagittal or coronal orientation before fixing in ice cold paraformaldehyde (PFA, 4% w/v in PBS) or methanol:acetic acid (3:1). Tissues were fixed overnight at 4°C.

PFA fixed brains were either embedded in wax or frozen in OCT. For wax embedding, PFA fixed brains were washed in PBS (1 h), dehydrated through an ethanol series (1 h each in 30%, 50%, 70%, 80%, 90%, 95%, 100% v/v), followed by isopropanol (1 h) and cleared in toluene (2 × 30 min). Methanol:acetic acid fixed tissues were washed in 70% ethanol and dehydrated and cleared as described above. Cleared brains were infiltrated with paraffin-wax at 58°C (Fibrowax™, VWR International, Leuven) (2 × 12–24 h). Brains were orientated in paraffin-wax filled moulds, allowed to set and blocks stored at 4°C until sectioned.

PFA fixed brains for cryosectioning were washed in PBS (1 h), transferred to sucrose solution (30% w/v in PBS with azide 0.05% w/v) and allowed to sink at 4°C (approximately 1–4 days). Brains were transferred to a 1:1 solution of sucrose:OCT™ (Optimal Cutting Temperature Compound, VWR, UK) at 4°C (30 min). Tissues were placed in moulds containing OCT™ and frozen on a metal plate cooled on dry ice. Once frozen, tissue blocks were stored at −80°C until sectioned.

### Sectioning of tissues

Paraffin sections (10 μm) were cut using a microtome (Leica Jung RM2035). Sections were floated on warm water (40°C), mounted on subbed slides, dried overnight (40°C) and stored at 4°C. Frozen OCT embedded brains were cryosectioned (20 μm) using a cryostat (Leica CM1850). Sections were pressed onto cold subbed slides and slowly warmed to room temperature. Sections were dried at room temperature (30–60 min) and stored at −20°C. Haematoxylin and eosin staining

Sections of paraffin embedded brains were dewaxed in Histoclear™ (National Diagnostics, Yorkshire) or Xylene (2 × 5 min) and rehydrated through decreasing ethanol series (2 × 3 min: 100%, 1 × 3 min: 95%, 75%, 50%, 30% v/v) with a final wash in water. They were immersed in Mayer’s Haematoxylin (5 min), rinsed in water (2 × 2 min), and treated with ammonia water (0.0005% v/v, 30 s) to develop the blue colour. Slides were washed in water (2 × 2 min), dehydrated through increasing ethanol series (2 min × 30%, 50%, 70%) and counterstained in Eosin-Y (1% w/v in 70% ethanol, 2 min) and rinsed rapidly though an ethanol series (3 s: 70%, 90%, 2 × 2 min: 100%), cleared in Histoclear™ or Xylene (2 × 5 min) and mounted with DePeX™ mounting medium (Gurr; VWR International, Leuven). Frozen OCT embedded sections were allowed to warm to room temperature, washed with PBS (5 min), water (5 min) and stained as described above.

### Immunohistochemistry

Sections of paraffin embedded brains were dewaxed in Histoclear™ or Xylene (2 × 5 min). Slides were rehydrated through decreasing ethanol series (2 × 5 min: 100%, 3 min: 95%, 75%, 50%, 30%), washed in water (5 min) and PBS (5 min). Frozen sections were warmed to room temperature and washed in PBS (5 min) to remove OCT. If required, antigen retrieval was carried out by placing sections in antigen retrieval solution (Vector Laboratories) and microwaved until boiling for 20 min, after which they were allowed to cool (20 min). After antigen retrieval, slides were briefly dried, sections circumscribed with ImmEdge PAP pen (Vector laboratories Ltd, UK) and incubated in blocking buffer at room temperature (1 h) followed by incubation with primary antibodies (see Supplementary Material, Table S2 for list of primary antibodies) overnight at 4°C. Slides were washed in PBS with Tween-20 0.1% (v/v) (PBST, 4 × 10 min). Appropriate secondary antibodies (Supplementary Material, Table S2) were used at a dilution of 1:1000 in blocking buffer and incubated for 1 h at RT. In all cases 4′,6-Diamidino-2-Phenylindole (DAPI) was also added to secondary antibody mixture (1 μg/ml). Slides were washed in PBST (2 × 10 min) followed by PBS (2 × 10 min) and coverslips mounted with Mowiol (Polysciences Inc., Germany).

### BrdU administration and detection

Bromodeoxyuridine (BrdU, 20 mg/ml in PBS) was administered to mice by intraperitoneal injection (100 mg/kg bodyweight) 1 h prior to euthanasia. Brains were dissected, fixed in methanol: acetic acid and processed for wax embedding. Paraffin sections were cut and dewaxed overnight in xylene and rehydrated. Sections were incubated in HCl (0.1 M) containing Pepsin (0.01% w/v) at room temperature (20 min). Slides were washed in PBS (2 × 10 min), slides briefly dried and sections circumscribed with PAP pen. BrdU incorporation was detected by immunohistochemistry using the monoclonal antibody G3G4 to BrdU (Supplementary Material, Table S2).

### Image acquisition and analysis

Brightfield images were acquired with DMRB microscope using Leica DFC490 camera and Leica Application Suite (LAS) software. Low power brightfield images were obtained with Leica WILD MZ8 stereomicroscope using Leica DFC490 camera. Fluorescence images were acquired with DM5500B microscope equipped with motorized stage and Leica DFC360FX camera. Depending on the tissue thickness or specific stain either single images or *Z*-stacks were acquired using LAS software. LAS 3D-deconvolution algorithm was applied to *Z*-stacks. Following deconvolution, either a single plane of focus was selected or *Z*-stacks were merged to create a maximum intensity projection. Comparisons were always made between images acquired and processed identically.

### Image processing

Fiji imaging software was used to estimate length, area and cell/cilia numbers (68). Data were subsequently exported to Microsoft Excel or Minitab17 for further analysis. Fiji software was also used to ‘stitch’ large composite pictures from overlapping fluorescence images. Sequential images of equal exposure were taken in a grid across the region of interest with adjacent images having a small overlap. Software aligned identical overlapping regions to stitch images together. Brightfield and fluorescence images were processed using Adobe Photoshop software to adjust brightness. Adjustments were always applied to whole images and equally between images to be compared.

### Statistical analysis

Statistical analysis was completed using Minitab17 software. Results were tested for normal distribution using Anderson Darling test and scores with *P* > 0.05 were deemed normal. The Bonett’s test, a modified version of Lavard’s test, and was used to determine variance of the mean and scores with *P* > 0.05 were deemed to have equal variance. Normal data with equal variance were analyzed using parametric tests; typically, a Student’s *t*-test was used for comparison of means. Data that did not have either a normal distribution or equal variances were transformed to improve the distribution. If transformation was successful, parametric analyses were completed on transformed data, however if data still violated these assumptions non-parametric analyses were completed; typically, a Mann–Whitney U test was used for comparison of median values.

### Quantification of primary cilia

The number of cells with primary cilia were identified by immunostaining for adenylyl cyclase III (ACIII). Nuclear DAPI labelling was used to quantify the total number of cells and the number of cells with a primary cilium expressed as a percentage. A region of interest (ROI) of 50 μm width was selected spanning a cross-section of both GCL and SGZ in the hippocampus. ROIs utilized the full thickness of the section (a *Z*-stack of 20 μm depth). Primary cilia were counted in ROIs from three sections of the brain per mouse and an average value was calculated. Average values from three mice were then used to make comparisons (*n* = 3). Data were normal with equal variance and a Student’s *t*-test was used to show significance between means.

### Analysis of Hippocampal thickness

The thickness of the GCL and SGZ was quantified by taking five equidistant measurements for each section and an average thickness obtained. Data from three brain sections from each mouse were used to calculate an average and three mice were used for comparisons (*n* = 3). Data were normal with an equal variance, so a Student’s *t*-test was used to compare mean values.

### Hippocampal cell type and analysis of proliferation

Hippocampal cell type and proliferation was quantified from sections co-immunostained for Paired box protein (Pax-6) and Neuronal nuclei (NeuN) or mini-chromosome maintenance protein 2 (MCM2) and marker of proliferation MKI67 (Ki67). The total number of labelled cells per 50 000 μm^2^ was counted in the GCL and SGZ. Data were acquired from three brain sections per mouse and used to calculate an average. Average values from three mice were used for comparisons (*n* = 3). All data were normal with equal variance and was compared using a Student’s *t*-test.

The proportion of Ki67 and MCM2 cells was compared by expressing the number of single- and double-labelled cells as a percentage of total labelled cells for each section. Data were acquired from three brain sections per mouse and used to calculate an average. Average values from three mice were used for comparisons (*n* = 3). Percentages were normal with equal distribution and Student’s *t*-test was used to compare mean values. Ki67-positive cells with GFAP-positive fibres were identified using high magnification images of individual cells through a 20 μm *z*-stack. The number of single- and double-positive cells were counted. The total number of labelled cells pooled to calculate the final percentage. Due to the low numbers of cells, statistical tests were not performed.

### Quantification of misplaced Hippocampal neurons

NeuN-positive neurons positioned outside of the dentate gyrus were identified in a ROI encompassing the entire length of the GCL and extending 50 μm into the ML. The number of NeuN-positive cells were quantified and expressed as number of cells per 1000 μm. Data were acquired from three brain sections per mouse and used to calculate an average. Average values from three mice were used for comparisons (*n* = 3). Data were normally distributed with equal variance and a Student’s *t*-test was used to compare means.

### Isolation of hippocampal cells for neurosphere culture

Five day old pups were generated by crossing *Talpid3fl/fl;UbcCreERT2* stud males to *Talpid3^fl/fl^* females. Brains were dissected from P5 mice and placed in DMEM/F-12 media (Life Technologies, UK). For generating hippocampal neurospheres, each brain was cut into coronal slices, transferred to DMEM/F12 media and dorsal hippocampi were isolated and incubated in 2 ml of digestion solution (1 mg/ml papain, 0.25 mg/ml L-cysteine, 1.1 mm EDTA,0.6% Glucose in PBS) at 37°C for 15 min.

Cell clumps were triturated into a single cell suspension, clumps were removed by passing through sieve (40 μm). Hippocampal single cells were centrifuged (840 × *g* for 8 min) and cell pellet was washed with DMEM/F-12 (6 ml). The cell suspension was centrifuged (840 × *g* for 8 min) and the cell pellet obtained from individual mouse hippocampus was re-suspended in neurosphere culture medium (1:1 DMEM/F12, 2% B27-VitaA, 2 μg/ml Heparin, 20 ng/ml hFGF2, 20 ng/ml hEGF, 0.5% w/v methyl cellulose) and plated in 3.5 cm bacteriological petri dishes (10 000 cell/cm^2^) in 2 ml neurosphere culture medium and incubated for 7 days at 37°C in 5% CO_2_.

The genotype of each neurosphere culture was determined by PCR of DNA isolated from the spare tissue of dissected individual brains from which the line was derived. Experimental data were acquired from three *Talpid3fl/fl;UbcCreER^T2^* hippocampal neurosphere lines isolated from three different mice (referred to as # 1, 2, 3).

### Passaging of cultured neurospheres

Neurospheres were passaged by pH-disassociation using alkaline DMEM/F12 (pH 11.5) every 7 days and single cells resuspended in fresh culture media. Cell suspension was neutralized with acidic DMEM/F-12 (pH 2.0) and passed through a sieve (40 μm) to get rid of cell clumps. Cell viability was determined by Trypan blue dye exclusion. Cells were centrifuged and cell pellets were re-suspended in neurosphere culture media and plated in bacteriological petri dishes or 96 well plates (5000 cells per cm^2^, typically a 1:10 split).

### Tamoxifen administration to cell cultures and genotyping to assess deletion of exons 11 and 12

All experiments for inducible deletion of *Talpid3* were performed on *Talpid3^fl/fl^* cells and *Talpid3fl/fl;UbcCreERT2* at passage 3. Disassociated neurospheres were plated in bacteriological dishes or 96-well tissue cultures plates in culture media containing 1 μm 4-hydroxytamoxifen (4-OHT) and this was followed by an additional dose of 1 μm after 3 days of growth.

DNA was isolated from neurospheres of each genotype either with or without tamoxifen administration using the HotSHOT cell lysis method (69). The neurosphere lines were genotyped for the *Talpid3^fl^* and *Talpid3* exon 11–12 deleted alleles and for *Cre* using primers listed in Supplementary Material, Table S1.

### Colony forming efficiency and colony size comparison

Twelve wells of a 96 well plate were plated with 1600 cells/well of each of the three independent *Talpid3fl/fl;UbcCreER^T2^* cell lines (#1, 2, 3) to compare the effect of deletion of *Talpid3* upon administration of Tamoxifen. This was followed by addition of media alone or media supplemented with tamoxifen. Media changes were made every third day. Control cells of genotype *Talpid3^fl/fl^* were also compared with and without tamoxifen administration.

For estimation of colony size, 7-day neurospheres cultured in petri dishes either with or without administration of tamoxifen were disassociated into single cells. After disassociation they were plated in 96-well tissue culture plate as described above without any further administration of tamoxifen. These are referred to as ‘passage +1’.

To measure colony size, a single image was acquired of a colony at the centre of each of the twelve wells using Leica DMIL microscope equipped with Leica EC3 camera. The area of every visible colony was measured using Fiji imaging software and all measurements taken from the 12 wells were pooled. Colony areas did not have a normal distribution and transformation was unable to normalize the data. Median values were compared using a Mann–Whitney U test.

### Neurosphere migration assay

Neurospheres of similar size were selected from 7-day cultures with and without 4-OHT treatment. Single neurospheres were plated in each well of a 24-well tissue culture dish coated with matrigel containing differentiation media (1 ml). Neurospheres were imaged after 2, 24, 48 and 72 h using Leica DMIL microscope equipped with Leica EC3 camera. The total neurosphere area was measured from images using Fiji imaging software. Twelve neurospheres from three independent lines of each genotype were used to make comparisons and results pooled between cell lines of the same genotype (*n* = 36). Data did not have an equal variance and could not be equalized by transformation, so a Mann–Whitney test was used to show significance between median values.

### Differentiation of neurosphere cells

Neurospheres were disassociated using the pH method. Cells were re-suspended in neurosphere culture media (2 00 000 cells/ml) and 500 μl added per well of Matrigel-coated coverslips in 24-well plate (50 000 cell/cm^2^). After 2 h, media was aspirated and replaced with differentiation media (500 μl, DMEM/F12 1:1, 2% B27-VitaA, 2 μg/ml Heparin, 2.5% v/v FGF2 and 2 ng/ml FGF2). After 3 days, media was aspirated and replaced with fresh differentiation media (500 μl). After 6 days, media was aspirated and cells washed with PBS (500 μl). PBS was aspirated and cells fixed on ice (30 min) with ice cold PFA (500 μl) or ice cold methanol:acetic acid (3:1, 500 μl). PFA-fixed cells were washed with PBS (2 × 10 min) and stored in PBS with sodium azide (0.05%, w/v) at 4°C. Methanol:acetic acid fixed cells were washed in 70% ethanol (2 × 10 min) and stored in 70% ethanol at 4°C.

### Immunohistochemistry of cultured cells

Fixed cells on coverslips were transferred into a fresh 24-well culture plate containing PBS (10 min). PBS was aspirated and PBT-block was added (500 μl, appendix 1.28). Cells were allowed to block at room temperature (1 h), block was aspirated and primary antibodies (see Table S2) diluted in PBT-block were added (100 μl). Primary antibodies were allowed to incubate overnight at 4°C. Primary antibodies were removed and PBST added (500 μl) to wash coverslips (10 min). PBST was aspirated and the wash repeated a further three times. PBST was aspirated and appropriate secondary antibodies (see Table S3) and DAPI diluted in PBT-block were added (100 μl). Coverslips were incubated at room temperature (1 h) after which secondary antibodies were removed. PBST was added (500 μl) to wash coverslips (10 min), PBST was aspirated and wash repeated once more. PBS was then added (500 μl, 10 min), PBS was aspirated and wash repeated once more. A small drop of Mowiol (~20 μl) was placed on a microscope slide and coverslips were mounted with cells facing down. Once dry, coverslips were sealed with nail varnish and imaged.

### Staining for F-actin with Phalloidin

F-actin was identified by following the initial steps of After the first PBT-block Phalloidin conjugated to fluorescence Alexa Fluor^®^ 488 Phalloidin (Life Technologies, UK) was diluted 5 μl per 200 μl of PBS with BSA (1% w,v) according to manufacturer’s instructions. DAPI was also added to the Phalloidin-488 solution, added to coverslips and incubated at room temperature (30 min). Coverslips were washed with PBS (4 × 10 min) and mounted with Mowiol.

### Quantification of ciliated cells

Neurosphere cultures plated on coverslips were immune-stained for adenylyl cyclase III and acetylated α-tubulin to identify primary cilia. Two coverslips from each cell line were stained and four random ROIs were imaged from each coverslip. The number of ciliated cells in each ROI was quantified and expressed as a percentage of total cell number. Values were pooled between cell lines and used for comparison between conditions (*n* = 24). Data were normally distributed with equal variance and a Student’s *t*-test was used to compare mean values.

## Supplementary Material

Supplementary_information_ddac095
