# Supplementary material for: Hippocampals neurogenesis is impaired in mice with a deletion in the coiled coil domain of Talpid3—implications for Joubert syndrome
Source: Hum Mol Genet. 2022 Apr 26;31(19):3245–65. doi: 10.1093/hmg/ddac095 (PMC9523558; doi:10.1093/hmg/ddac095)
Supplement: Supplementary_information_ddac095 [file supplementary_information_ddac095.docx]

**Supplementary Information**

Hippocampal neurogenesis is impaired in mice lacking the coiled coil domain of Talpid3

**A**ndrew L Bashford and Vasanta Subramanian*

| **Table S1 List of Primers for genotyping of mice** | | | |
| --- | --- | --- | --- |
| **Name** | **Sequence** | **Product** | **Reference** |
| Talpid3 (flox) | FP-5’ TGCCATGCAGGGATCATAGC 3’ | 470bp (fl), 351bp (wt) | (Bangs et al., 2011) |
|  | RP-5’ GCTAGTACATTGCTGCAAGC 3’ |  |  |
| Talpid3 (deleted) | FP-5’ TGCCATGCAGGGATCATAGC 3’ | 273bp | (Bangs et al., 2011) |
|  | FP-5' GAGCACACTGGAGGAAAGC 3’ |  |  |
| Cre | FP 5’CATTACCGGTCGATGCAACGAGTGATGAG3’ | 408bp | Subramanian Lab |
|  | RP 5’GAGTGAACGAACCTGGTCGAAATCAGTGCG 3’ |  |  |

| **Table S2. List of primary antibodies used for immunohistochemistry** | | | | |
| --- | --- | --- | --- | --- |
| **Target** | **Host** | **Dilution** | **Supplier** | **Code** |
| Adenylyl cyclase III | rabbit | 1:1000 | Santa Cruz | C-20 |
| Acetylated α-tubulin | mouse | 1:1000 | Sigma-Aldrich | T6793 |
| Beta III tubulin | mouse | 1:500 | Chemicon | MAB1637 |
| BLBP | rabbit | 1:500 | AbCam | Ab32423 |
| BrdU | mouse | 1:50 | DSHB | G3G4 |
| Dcx | rabbit | 1:200 | Cell signal | 460 4S |
| gamma-tubulin | rabbit | 1:1000 | Sigma-Aldrich | T5192 |
| GFAP | mouse | 1:400 | Sigma-Aldrich | G3893 |
| GFAP | rabbit | 1:100 | Invitrogen | 180063 |
| Gli1 | rabbit | 1:500 | Cell Signal | V812 |
| Ki67 | rabbit | 1:100 | Millipore | Ab9260 |
| MCM2 | goat | 1:100 | Santa Cruz | SC9839 |
| Nestin | mouse | 1:50 | DSHB | Rat401 |
| NeuN | mouse | 1:1000 | Millipore | Mab377 |
| Neurofilament | mouse | 1:5 | DSHB | 2H3 |
| Pax6 | rabbit | 1:500 | Covance | PRB-278P |
| PCNA | mouse | 1:4000 | Cell signalling | 2586 |
| Pericentrin | rabbit | 1:2000 | AbCam | ab4448 |
| Phosphohistone 3 | rabbit | 1:1600 | Cell Signal | 3377 |
| Sox2 | rabbit | 1:500 | Millipore | Ab5603 |
| Tbr2 | rabbit | 1:500 | AbCam | Ab23345 |

| **Table S3 List of secondary antibodies used for immunohistochemistry**. All secondary antibodies were used at 1:1000 dilution. † and * indicate the most commonly used dual labelling pairs. | | |
| --- | --- | --- |
| Fluorophore name | Specificity | Molecular Probes® cat # |
| Alexa Fluor 488* | Goat anti-Mouse | A-11001 |
| Alexa Fluor 488† | Goat anti-Rabbit | A-11034 |
| Alexa Fluor 488 | Goat anti-Guinea Pig | A-11073 |
| Alexa Fluor 488 | Donkey anti-Goat | A-11055 |
| Alexa Fluor 594* | Goat anti-Rabbit | A-11012 |
| Alexa Fluor 594 | Donkey anti-Goat | A-11058 |
| Alexa Fluor 568† | Goat anti-Mouse | A-11004 |
| Alexa Fluor 568 | Rabbit anti-Mouse | A-11061 |
| Alexa Fluor 488 | Goat anti-Rat | A-11006 |

Supplementary Figures


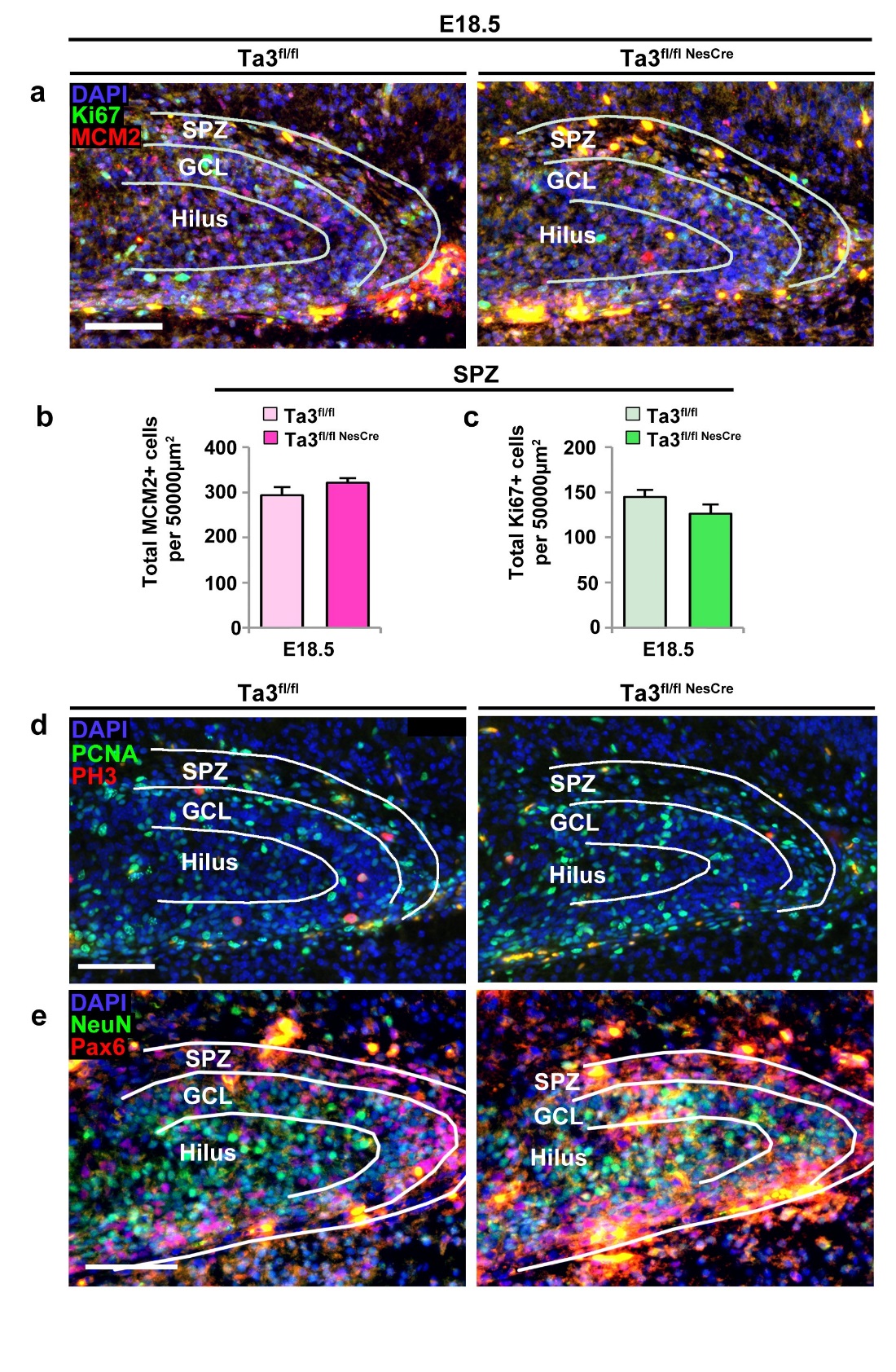


**Supplementary Figure 1. Dentate gyrus appears normal in E18.5 *Talpid3* mutant mice**

(a) E18.5 dentate gyrus immuno-stained for Ki67 (green) and MCM2 (red). (b) Quantification of total MCM2+ progenitors in the subpial zone (SPZ). (c) Quantification of total Ki67+ progenitors in the SPZ. Mutants (Ta3^fl/fl^NesCre) show no differences in the number of MCM2+ or Ki67+ progenitors. (d) E18.5 dentate gyrus labelled for PCNA (green) and PH3 (red). (e) E18.5 dentate gyrus labelled for NeuN (green) and Pax6 (red). Control (Ta3^fl/fl^) and mutant (Ta3^fl/fl^NesCre) show a comparable number and distribution of proliferating cells, progenitors and mature neurons. GCL, granule cell layer. Scale bar: 75µm (a,e). Error bars: (b) s.e.m (n = 3), Student’s *t*-test.


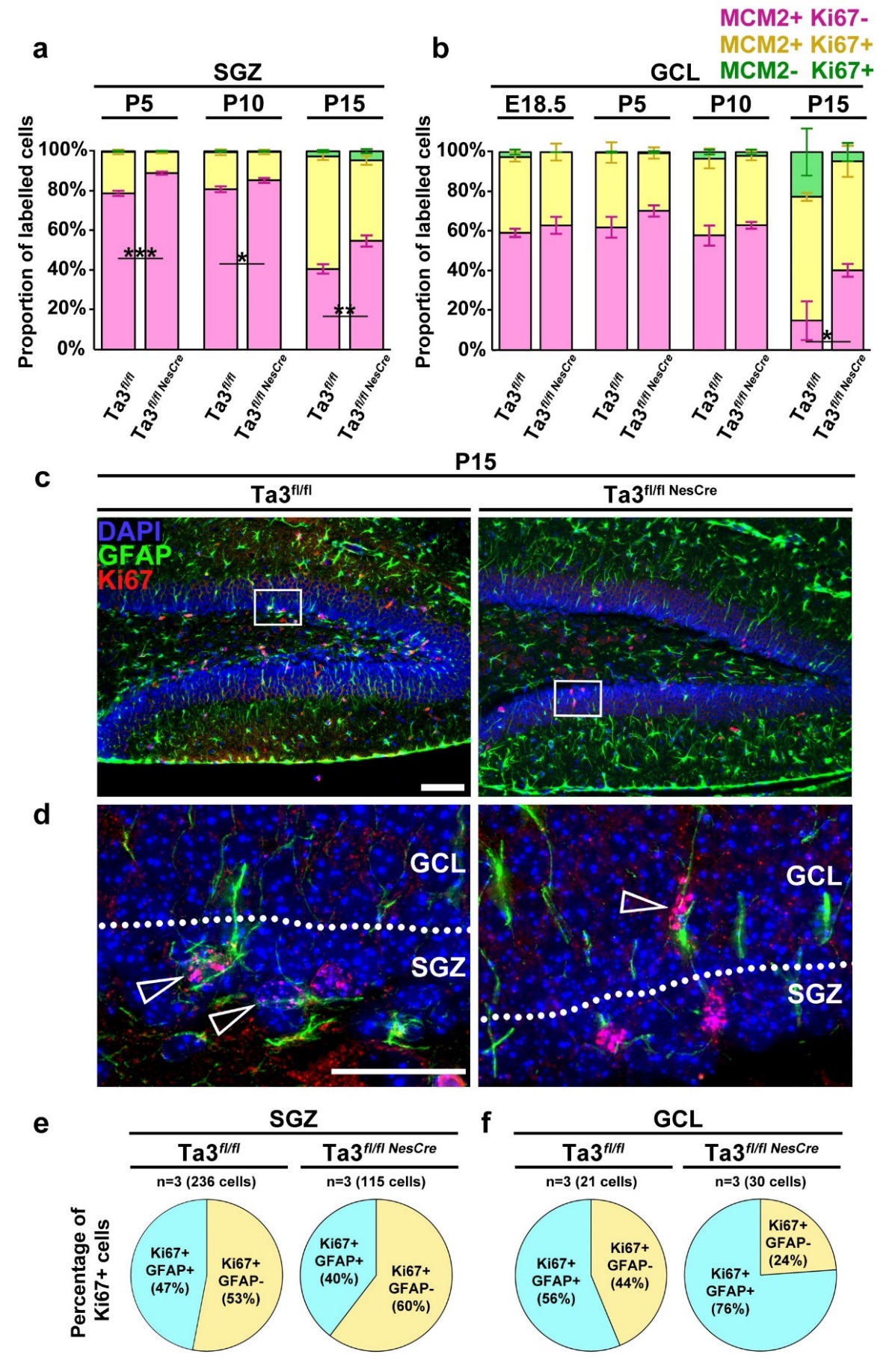


**Supplementary Figure 2. *Talpid3* Mutants have a lower proportion of proliferating progenitors**

(a) Proportion of cells labelled for MCM2, Ki67 or both in the SGZ. At P5, P10 and P15 Mutants (Ta3^fl/fl^NesCre) show a significant increase in the proportion of MCM2-positive progenitors which are Ki67 negative (red). (b) Proportion of cells labelled for MCM2, Ki67 or both in the GCL. At E18.5, P5 and P10 mutants (Ta3^fl/fl^NesCre) have similar proportions of progenitors in the GCL. At P15 control (Ta3^fl/fl^) more proliferating cells which are MCM2 negative (green). Mutants (Ta3^fl/fl^NesCre) show a distribution similar to that seen in the SGZ. (c) P15 dentate gyrus labelled for GFAP (green) and Ki67 (red). White box indicates region of higher magnification shown in (d). (e) Proportion of Ki67-positive cells which are likely to be RGC-progenitors (Ki67-positive, GFAP-positive, blue) in the P15 SGZ. Control (Ta3^fl/fl^) and mutant (Ta3^fl/fl^NesCre) have a similar proportion of cells. (f) Proportion of RGC-progenitors (Ki67-positive, GFAP-positive, blue) in the GCL. Mutants (Ta3^fl/fl^NesCre) have a greater proportion of RGC-progenitors. Due to low cell numbers in the control (Ta3^fl/fl^), statistical tests were not performed. Scale bar: 75µm (a-c). Error bars: (a,b) s.e.m (n = 3), * P≤0.05, ** P≤0.01, *** P≤0.001, Student’s *t*-test.


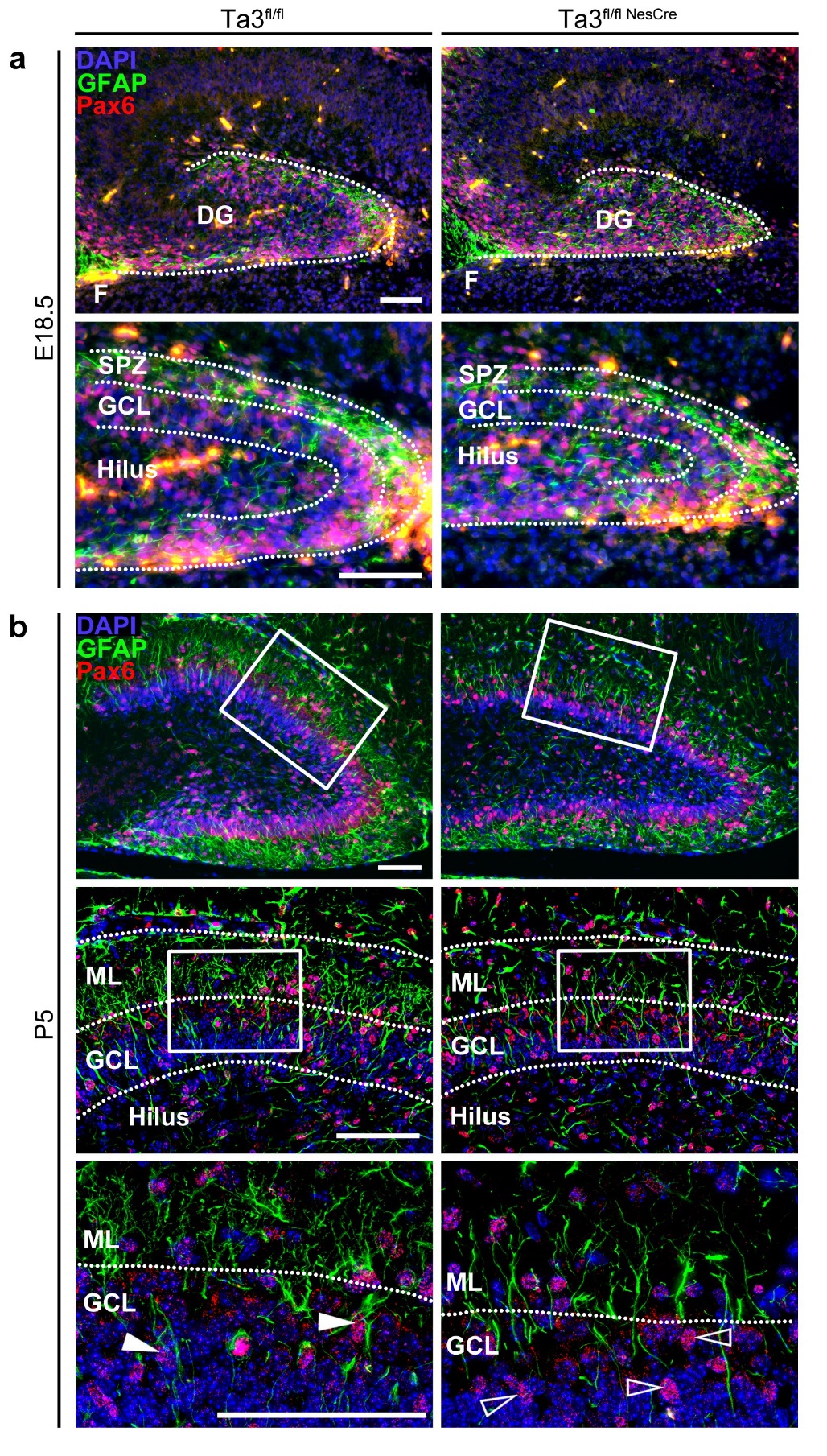


**Supplementary Figure 3. Fewer progenitors are associated with GFAP fibres in P5 *Talpid3* mutant hippocampus**

(a) E18.5 dentate gyrus labelled for GFAP (green) and Pax6 (red). Both control (Ta3^fl/fl^) and mutant (Ta3^fl/fl^NesCre) have dense GFAP+ fibres in the SPZ (b) P5 dentate gyrus labelled for GFAP and Pax6. The mutant (Ta3^fl/fl^NesCre) DG shows a striking reduction in branches throughout the ML with fewer progenitors associated with glial fibres. White boxes indicate regions of higher magnification. Progenitors associated with glial fibres are indicated by white arrows and without glial fibres are indicated by hollow arrows. Scale bars: 75µm (a-b).


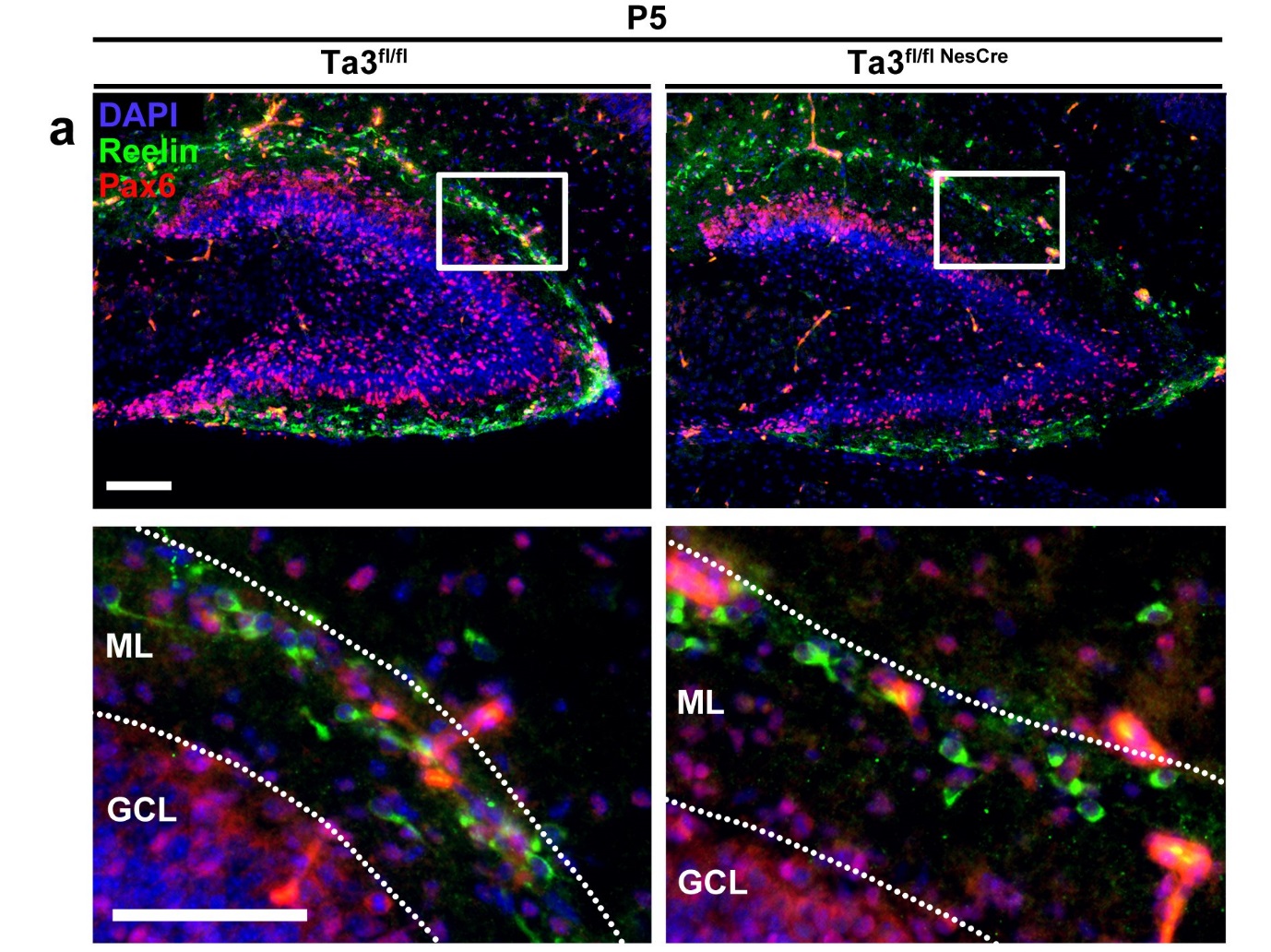


**Supplementary Figure 4. *Talpid3* mutant dentate gyrus shows slight reduction in Reelin producing cells**

(a) P5 dentate gyrus labelled for Reelin (green) and Pax6 (red). Both control (Ta3^fl/fl^) and mutant (Ta3^fl/fl^NesCre) show a band of Reelin-positive cells lining the outer edge of the ML. Mutant (Ta3^fl/fl^NesCre) Reelin+ cells have a similar morphology but appear to be fewer in number. Lower panel shows higher magnification of boxed area. Scale bar: 75µm (a).


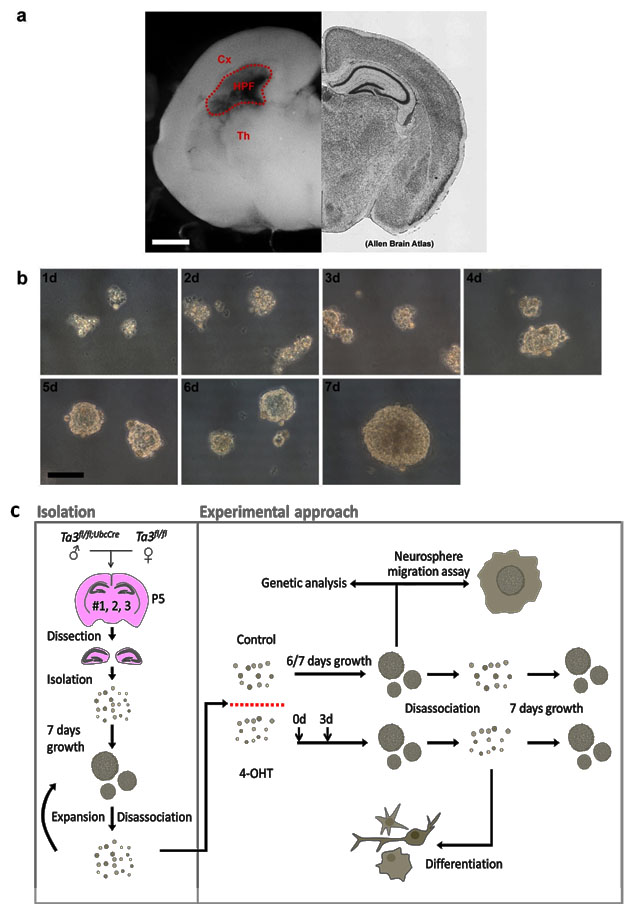


**Supplementary Figure 5 Experimental plan for generation of hippocampal neurosphere cultures**

a) Left: Image of coronal brain slice following removal of dorsal hippocampal formation (HPF), Right: representative coronal brain slice taken from Allen Brain atlas. (b) Representative images of isolated cells forming neurospheres throughout seven day growth period. (c) Schematic diagram showing isolation and experimental approach. Isolation: cells were isolated from P5 dorsal HPF and grown for seven days. Cell numbers were expanded by disassociation into single cells followed by further growth into neurospheres. Experimental approach: Neurospheres were disassociated into single cells, separated and recombination induced by administration of tamoxifen (4-OHT) immediately (d0) and after three days (d3) after plating. After 6/7 days growth neurospheres were used for genotyping, colony growth assay, colony forming assay migration assay or differentiated for 6 days. Abbreviations: Cx, Cortex; Th, Thalamus. Scale bar: 1 mm (a), 100 µm (b). Image taken from Allen Brain Atlas (www.brain-map.org).


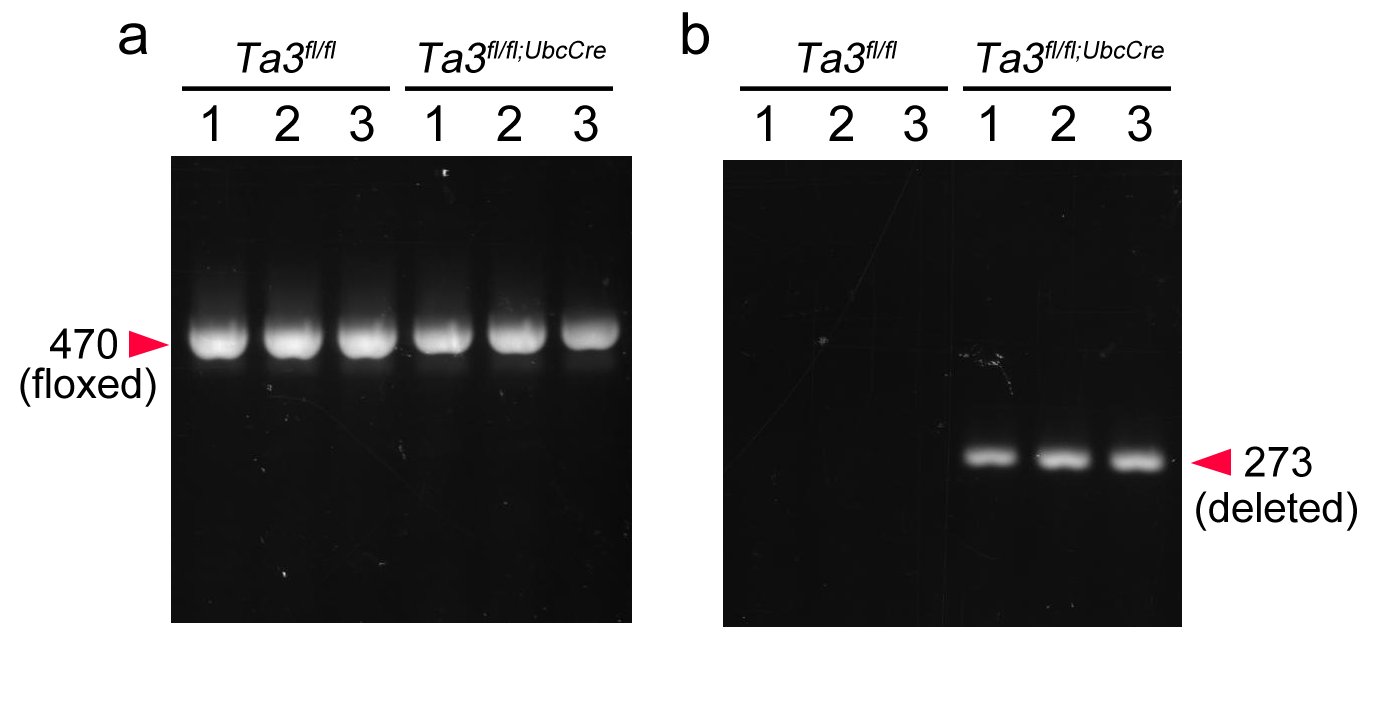
Figure 6

**Supplementary Figure 6. Genotypes of Ta3^fl/fl^UbcCre^T2^ neurospheres**

(a) Genotype of Ta3^fl/fl^ and Ta3^fl/fl^UbcCre^T2^ neurospheres without 4-OHT treatment. (b) Genotype of Ta3^fl/fl^ and Ta3^fl/fl^UbcCre^T2^ neurospheres with 4-OHT treatment.


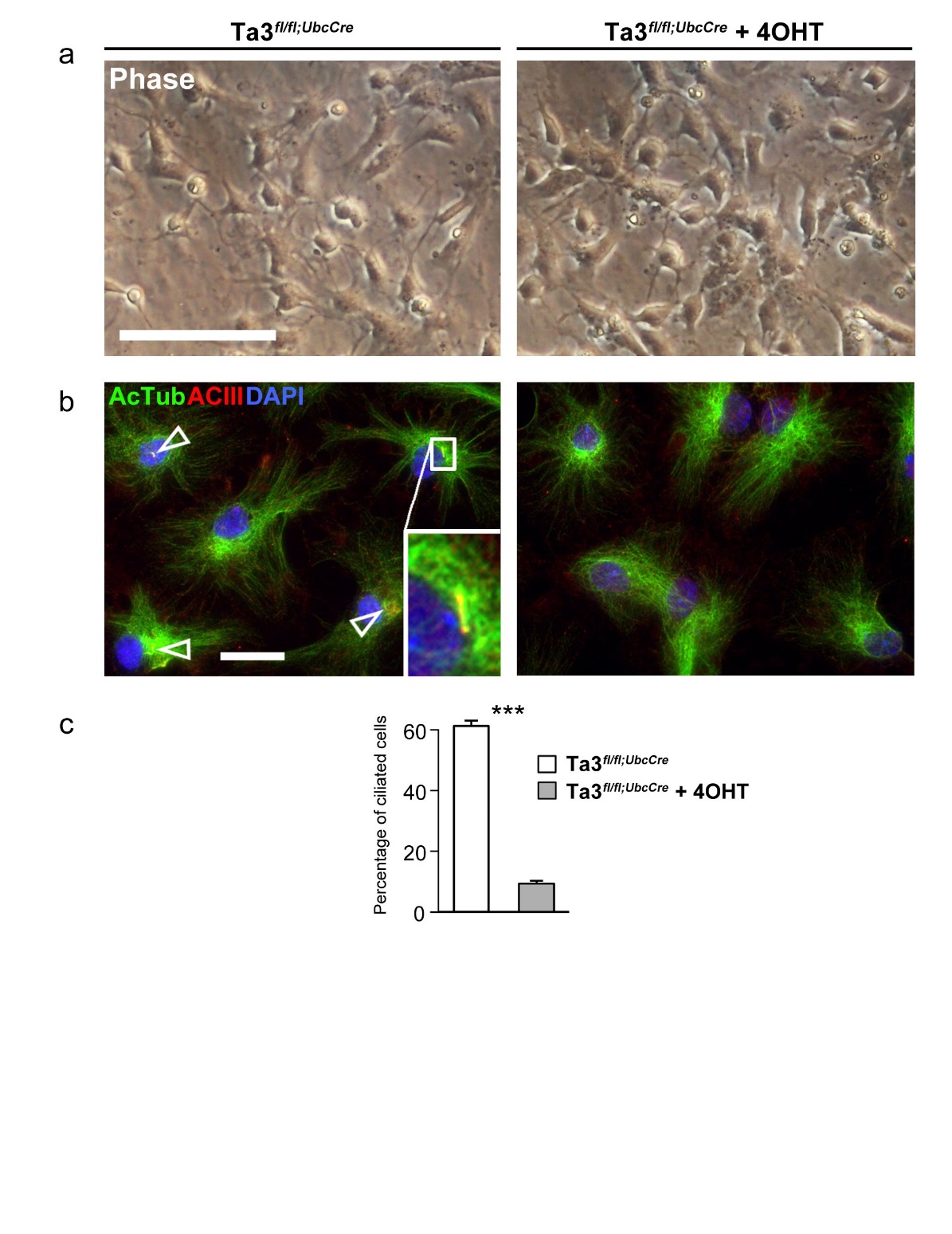


**Figure 7. *Talpid3* mutant differentiated neurosphere cells lack primary cilia**

(a) Representative phase contrast images of disassociated neurosphere ells differentiated on Matrigel for 24 hours with and without tamoxifen administration (b) Cells differentiated for 6 days immunolabelled for adenylyl cyclase III (red) and acetylated α-tubulin (green). Cultures derived from *Talpid3* mutant neurospheres show loss of primary cilia but with no apparent difference in microtubule organisation. (c) loss of *Talpid3* causes a significant reduction in the percentage of cells with primary cilia. Scale bar: 25 µm (a, b). Error bars (c) s.e.m (n = 12) *** P≤0.001, (one tailed Student’s *t*-test).


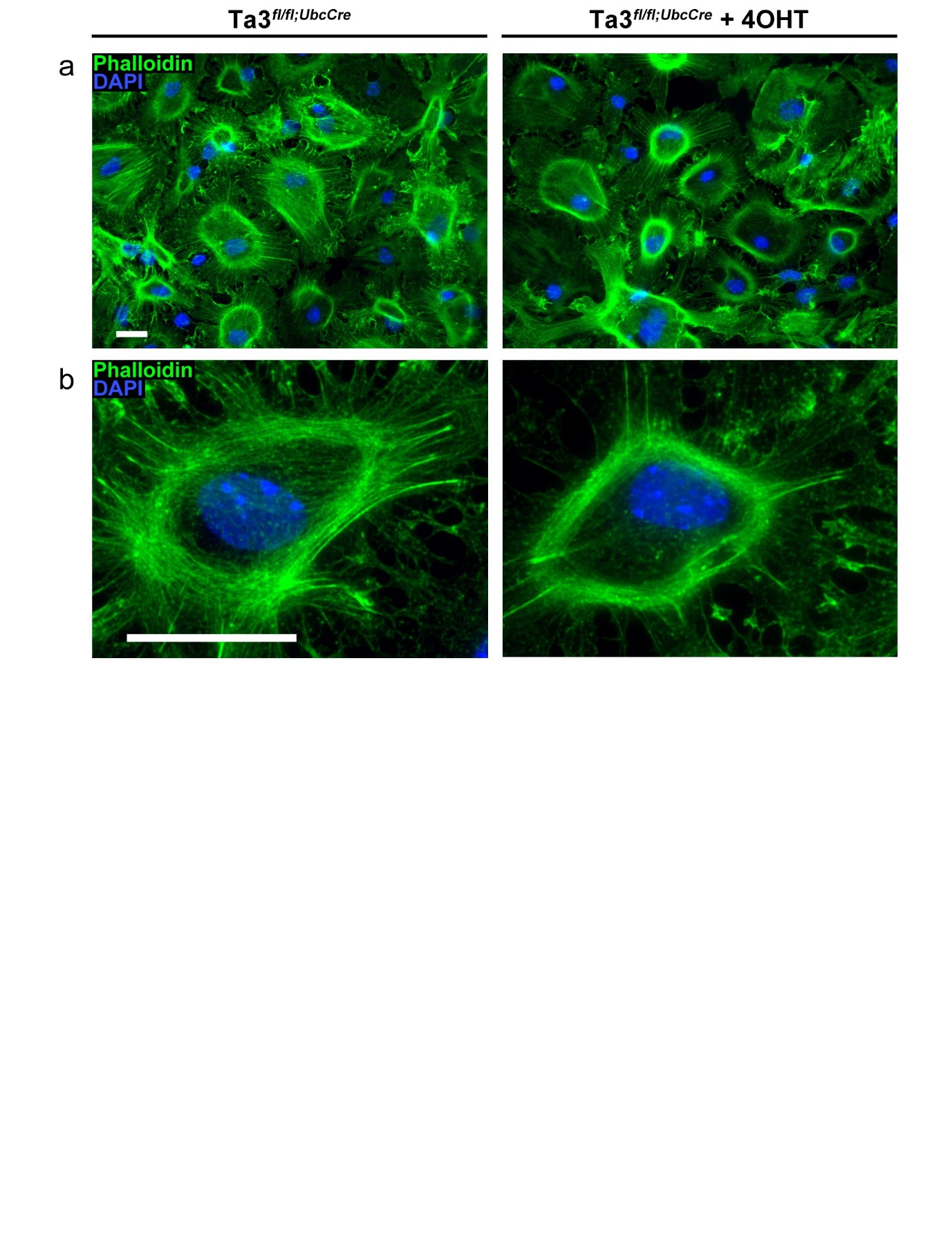


**Supplementary Figure 8. Deletion of *Talpid3* exons 11-12 leads to a reduction in F-actin stress fibres**

(a, b) Identification of F-actin organization using fluorescently labelled Phalloidin (green). Deletion in *Talpid3* causes a disruption of actin cytoskeleton with fewer stress fibres. Scale bar: 25 µm (a, b).
